# Extensive cross-regulation of post-transcriptional regulatory networks in *Drosophila*

**DOI:** 10.1101/gr.182675.114

**Published:** 2015-11

**Authors:** Marcus H. Stoiber, Sara Olson, Gemma E. May, Michael O. Duff, Jan Manent, Robert Obar, K.G. Guruharsha, Peter J. Bickel, Spyros Artavanis-Tsakonas, James B. Brown, Brenton R. Graveley, Susan E. Celniker

**Affiliations:** 1 Department of Biostatistics, University of California Berkeley, Berkeley, California 94720, USA;; 2 Department of Genome Dynamics, Lawrence Berkeley National Laboratory, Berkeley, California 94720, USA;; 3 Department of Genetics and Genome Sciences, Institute for Systems Genomics, University of Connecticut Health Center, Farmington, Connecticut 06030, USA;; 4 Department of Cell Biology, Harvard Medical School, Boston, Massachusetts 02115, USA;; 5 Biogen Incorporated, Cambridge, Massachusetts 02142, USA;; 6 Department of Statistics, University of California Berkeley, Berkeley, California 94720, USA

## Abstract

In eukaryotic cells, RNAs exist as ribonucleoprotein particles (RNPs). Despite the importance of these complexes in many biological processes, including splicing, polyadenylation, stability, transportation, localization, and translation, their compositions are largely unknown. We affinity-purified 20 distinct RNA-binding proteins (RBPs) from cultured *Drosophila melanogaster* cells under native conditions and identified both the RNA and protein compositions of these RNP complexes. We identified “high occupancy target” (HOT) RNAs that interact with the majority of the RBPs we surveyed. HOT RNAs encode components of the nonsense-mediated decay and splicing machinery, as well as RNA-binding and translation initiation proteins. The RNP complexes contain proteins and mRNAs involved in RNA binding and post-transcriptional regulation. Genes with the capacity to produce hundreds of mRNA isoforms, ultracomplex genes, interact extensively with heterogeneous nuclear ribonuclear proteins (hnRNPs). Our data are consistent with a model in which subsets of RNPs include mRNA and protein products from the same gene, indicating the widespread existence of auto-regulatory RNPs. From the simultaneous acquisition and integrative analysis of protein and RNA constituents of RNPs, we identify extensive cross-regulatory and hierarchical interactions in post-transcriptional control.

Gene expression involves a complex and often dynamic interplay between proteins and RNA. The synthesis and function of almost all known RNAs involve the formation of ribonucleoprotein particles (RNPs) (Draper 1995). These RNP complexes range from small (e.g., Cas9 bound to a guide RNA) to large (e.g., the ribosome or spliceosome). Very few RNP complexes have been characterized in any organism.

The protein components of RNPs can either interact directly with RNA through one or more RNA-binding domains or can be associated indirectly through interaction with another protein that is itself directly bound to RNA (Glisovic et al. 2008). Proteins such as NOVA2, PTBP1, U2AF2, and RBFOX2, as well as others, contain RNA-binding domains that directly bind RNA in a largely sequence-specific manner (Lewis et al. 2000; Jin et al. 2003; Kielkopf et al. 2004; Hall et al. 2013). In contrast, SMN, which is involved in snRNP biogenesis, lacks any known RNA-binding domains, and associates with the U snRNAs indirectly. Many assays characterizing protein–RNA interactions utilize UV-crosslinking to ensure that the observed interactions either are direct or occurred in cells prior to lysis (Mili and Steitz 2004). Though powerful, these approaches also have the following limitations. First, many RBPs that interact directly with RNA cannot be crosslinked to RNA due to the configuration of the RNA–protein interaction. Second, even for proteins that can be crosslinked to RNA, the efficiency of crosslinking is low and not every site of interaction is amenable to crosslinking. Finally, these approaches cannot capture indirect interactions, including proteins that are part of an RNP that do not directly contact RNA. Thus crosslinking-independent approaches are necessary to capture the larger RNA–protein interaction landscape.

In addition to the diversity of capture approaches used to study RNA–protein interactions, there are differences in the assays used to characterize the interacting molecules. Several groups have used probes to purify specific target RNAs and then identify the associated proteins, though these approaches often require tagging the target RNA (for review, see McHugh et al. 2014). Hentze (Strein et al. 2014) and Parker (Mitchell et al. 2013) have used oligo-dT to globally purify human and yeast cellular mRNA–protein complexes (mRNPs), respectively, and then identified the bound proteins but not the associated RNAs. However, very few studies have purified native RNP complexes and characterized both the RNA and protein components.

RNA-binding proteins (RBPs) play a crucial role in cellular biology, particularly in higher eukaryotic organisms, where ∼3% of genes encode proteins that have either known or predicted RNA-binding domains (Glisovic et al. 2008). RBPs participate in many essential post-transcriptional functions, including pre-mRNA splicing, 3′ end formation, RNA localization, turnover, and translation. Many RBPs participate in several of these processes (Glisovic et al. 2008). One example of a pleiotropic RBP is the Fragile X Mental Retardation Protein (FMR1), encoded in *Drosophila melanogaster* by *Fmr1*. FMR1 forms a complex with components of the RNAi machinery, including Argonaute 2 (AGO2), an essential component of the RNA-induced silencing complex (RISC) (Ishizuka et al. 2002). FMR1 also associates with the ribosome to directly block translation by inhibiting tRNA association (Chen et al. 2014a) and, in yet another capacity, functions as a translational activator (Bechara et al. 2009). Other proteins that have been shown to have pleiotropic effects include NOVA2 (Licatalosi et al. 2008), MBNL (Han et al. 2010) family proteins, and HNRNPH1 (Katz et al. 2010). It is likely that the participation of RBPs in multiple post-transcriptional processes will be common.

RBPs recognize their RNA targets through RNA-binding domains. In *Drosophila*, and most eukaryotes, common classes of RNA-binding domains include the RNA-recognition motif (RRM), the K homology domain (KH), the double-stranded RNA-binding motif (dsRBM), and zinc-finger motifs. As with transcription factors, there is no one-to-one mapping between domains and functional roles, and many RBPs with characterized functions appear pleiotropic. Some RBPs have strong sequence specificity for cognate binding sites, including NOVA2 and the fly homolog PS, which bind to YCAY repeats in species from insects to mammals, although the RNA targets regulated by NOVA2 and PS have changed substantially across metazoans (Buckanovich and Darnell 1997; Brooks et al. 2011). The RNAcompete assay has been used to identify in vitro binding specificities and relative affinities for a number of RBPs in several species (Shepard and Hertel 2009). A number of factors have been studied in vivo but largely within small-scale studies (e.g., Hogan et al. 2008; Zhao et al. 2010; Brazao et al. 2012; Chen et al. 2014b; Lu et al. 2014). An in vivo study in yeast (Hogan et al. 2008) surveyed the binding patterns of 40 RBPs and concluded that the targets of different factors fall into distinct functional classes, indicating that specific RBPs participate in defined regulatory pathways. A study of six of the seven *Drosophila* small RNP proteins (Sm proteins) showed that the Sm RNA targets fall into three categories: small nuclear RNAs (snRNAs), small Cajal bodies (scaRNAs), and mRNAs (Lu et al. 2014). The extent to which in vitro binding affinity models are sufficient to explain in vivo patterns of binding is unclear. In most cases, it is also largely unknown whether RBPs tend to bind RNA individually as monomers or in larger complexes.

To explore the compositions of RNPs in *Drosophila*, we characterized the RNA and protein components of RNPs purified using 20 distinct proteins as baits. These proteins were chosen based on their known RNA-binding domains (e.g., KH, RRM) or roles in RNA biology. We group these 20 RBPs into broad functional classes: Exon Junction Complex (EJC), which marks the location of splicing events and provides a link to processing events downstream from splicing, including nonsense-mediated decay (NMD; encoded by the release factor, *Upf1*) (Le Hir et al. 2001; Kurosaki and Maquat 2013); serine-arginine (SR) splicing factors that although primarily implicated in splicing have also been shown to participate in other post-transcriptional events (encoded by *B52*, *Rbp1*, *SC35*, *SF2*, *Srp54*, *tra2*) (Shepard and Hertel 2009); spliceosome-associated factors that interact with the canonical spliceosome complex (encoded by *snRNP-U1-70K*: abbreviated here as *snRNP70K*, *CG6227*, *Cbp20*, *Rm62*, *U2af50*) (Sabin et al. 2009; Will and Luhrmann 2011); heterogeneous nuclear ribonuclear proteins (hnRNPs), a functionally diverse group of proteins that participate in nuclear RNA processing and export yet contain no common domains (Han et al. 2010) (encoded by *elav*, *ps*, *mub*, *msi*, *Syp*); and, lastly, pleiotropic proteins, including factors with diverse functions in translational regulation and RNA localization (encoded by *Fmr1*, *qkr58E-1*, *qkr54B*).

A unique aspect of this study is that RNA and protein are copurified from the same IP reaction, an aspect of this study that is not possible in CLIP-seq or other crosslinking-dependent procedures. We utilize RIP to identify both the RNA and protein components of ribonuclear complexes. Analysis of the RNAs and proteins associated with these RBPs reveals a densely interconnected network of interactions. Many RBPs associate with the RNA and protein products encoded by the same gene and, therefore, may regulate both the protein and RNA components of dozens of RNP complexes. More generally, the RNAs encoding proteins involved in post-transcriptional regulation tend to be bound by most of the factors in our study, forming “high occupancy targets” (HOT) RNAs. Several studies (e.g., Kosti et al. 2012) have shown that the RNAs encoding RBPs tend to be post-transcriptionally regulated, suggesting that this may occur more often for post-transcriptional regulators than other types of genes. Our data reveal that this tendency may derive from local interactions in the regulatory network, where RBPs interact with and presumably regulate the mRNAs encoding their protein interaction partners. Hence, via the integrative analysis of matched protein and RNA interaction data, we identify a poorly studied layer of feedback in the hierarchy of gene regulation of metazoan cells.

## Results

### Identification of the RNA and protein components of RNP complexes

**Figure 1.**
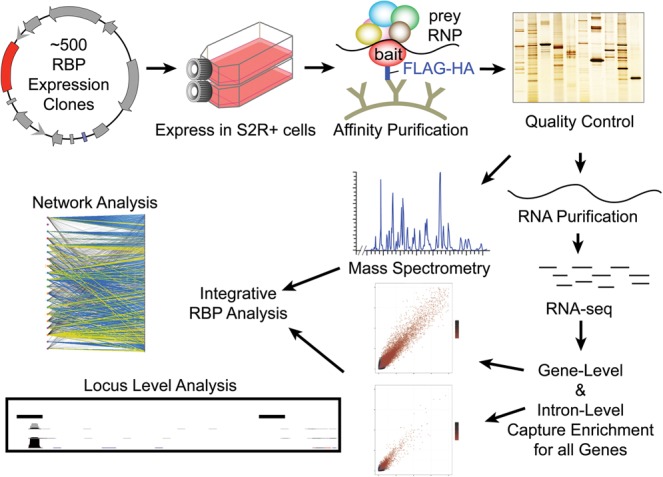
Data production and processing. The data processing pipeline is described here, starting from transfection of RNA-binding proteins into S2R+ cells. Immunoprecipitation is then performed to pull down ribonucleoprotein particles (RNPs). The protein and RNA components of the RNPs are then separated and measured with MS/MS and RNA sequencing. These data are then analyzed together at global and local levels.

To explore the composition of RNP complexes in *Drosophila*, we purified RNP complexes under native conditions (without crosslinking) from cultured cells expressing 20 different epitope-tagged RBPs and then analyzed the protein components by mass spectrometry and the RNA components by RNA sequencing (Fig. 1). The proteins selected for these experiments were chosen because they contained KH- or RRM-type RNA-binding domains or DEAD-box RNA helicase domains or because they lacked known RNA-binding domains but have important roles in RNA biology (Supplemental Table 2). The proteins studied are known to function in splicing, NMD, translation regulation, and RNA localization and include members of the SR and hnRNP families of proteins, core components of the spliceosome, and components of the EJC (Supplemental Table 2). For each protein, we added a C-terminal Flag-HA epitope to the longest ORF in a vector that allowed inducible expression in transiently transfected cells. This is the same strategy that was developed and demonstrated to be highly effective to produce a *Drosophila* Protein Interaction Map (DPiM) (Guruharsha et al. 2011).

Tagged RBPs were transfected into *Drosophila* S2R+ cells in biological duplicate. We used as controls both empty vector and four different non-RBPs. For these experiments, cell lysates were prepared in the presence of RNase inhibitors to maintain an RNase-free environment to facilitate recovery of intact RNAs. The RNA–protein complexes were purified by immunoprecipitation (IP), and the products of each co-IP were split into two equal fractions. One fraction was used for LC/MS/MS analysis to define the protein composition of the sample and relate the proteins to the DPiM, and the second fraction was depleted of rRNAs and subjected to RNA sequencing to analyze the associated polyadenylated and nonpolyadenylated RNAs.

This experimental approach results in the identification of protein–RNA (RIP-seq) and protein–protein (MS/MS) interactions in a single IP reaction. Because we do not crosslink, we pull down whole complexes, and therefore, our data do not distinguish between direct and indirect interactions or binding events. When we identify interactions of two RBPs with mRNAs from the same gene, we conclude that these two factors share a common target, though the protein–RNA interactions can occur on either the same or different mRNA molecules. However, if we additionally observe protein–protein interactions between these RBPs, we conclude that there is evidence for the existence of an RNP complex that includes the target RNA and the two RBPs. RBPs interact with many RNAs and proteins present in S2R+ cells. Hence, our data are amenable to network analysis techniques that identify community structure. Because we observe whole complexes, not individual pairwise interactions, we expect stable RNPs to yield densely connected “cliques” of associated RNA and protein molecules. Our data are consistent with this model and are described as follows.

To identify RNAs enriched by each RBP, we mapped sequenced reads to the genome and then quantified the capture level (analogous to expression level in a knockout experiment) of each gene (FlyBase r5.57) in both the IP and control experiments with DESeq (Methods) (Anders and Huber 2010). We applied two thresholds to the DESeq output: a local irreproducible discovery rate (IDR) of 10% (∼3.2% global IDR; Methods) and a fold change (FC) of 50% in both biological replicates, corresponding to a local signal to noise ratio of at least 2.0. The IDR, a standard technique for the analysis of IP data developed by the ENCODE Project Consortium, is analogous to the false-discovery rate (FDR) and leverages biological replicates to measure quantitative reproducibility (Li et al. 2011; Landt et al. 2012). At this stringent cutoff, we recover an average of 1231 interacting RNAs per RBP (Supplemental Fig. 1; Supplemental Tables 3, 4). The RBPs we surveyed collectively show statistically significant enrichment of RNA products of 72% of genes expressed in S2R+ cells (Methods) and 40% of all genes in *Drosophila*.

As one way to assess the quality of our data, we examined our results for known RNA–protein interactions. For example, *snRNA:U1:82Eb* and *snRNA:U1:95Cc* are the two RNAs most strongly enriched by SNRNP70K, an integral component of the U1 snRNP. Consistent with the known interactions between the cap-binding complex and U snRNAs (Lewis et al. 1996), CPB20 interacts strongly with the U1, U4, U5, U11, and U12 snRNAs. Moreover, as *Rbp1* is known to cross-regulate *Rbp1-like* (Kumar and Lopez 2005), we observe a strong interaction of RBP1 protein with *Rbp1-like* mRNA. Thus, our data set recapitulates known protein–RNA interactions reported in the literature.

The majority of the factors in our study are involved in splicing regulation. In *Drosophila*, 74% of genes produce spliced transcripts (87% of genes expressed in S2R+ cells). Supplemental Table 5 shows that all but one RBP (CBP20, a component of the nuclear cap-binding complex) show strong enrichment for spliced genes (*P* < 0.005). Hence the preference of most RBPs in this study to bind spliced RNAs supports their functional roles as splicing regulators.

### HOT RNAs are a feature of post-transcriptional regulation

Most of the RBPs in our study associate with overlapping sets of target RNAs. A total of 74% (141 out of 190) of pairwise intersections of RBP target RNAs across all pairs are larger than expected at random (hypergeometric *P* < 0.01) (Supplemental Table 6). For example, *Smg5* mRNA, which encodes an RBP involved in NMD, interacts with RNP complexes containing 15 of the 20 studied RBPs. Indeed there are six such mRNAs (*CG12065*, *CG3008*, *CG7456*, *Hsp26*, *Hsp27*, and *Smg5*), which is considerably more than expected under an independence model (probability that the RNA bound by the most RBPs ≥15 is <0.001). The RNAs encoded by 282 genes (Supplemental Table 7) interact with half or more of the RBPs in our study (Supplemental Table 8), and we will refer to these RNAs as “high occupancy target” (HOT) RNAs. Under a model conservatively conditioned on the assumption that only RNAs associated with at least one RBP are available for binding, this constitutes 282-fold enrichment over expectation (Poisson-binomial *P* < 10^−15^). This threshold ensures that HOT RNAs are the targets of a diverse group of RBPs, including multiple binding domains and functional families. We note that the *qkr58E-1* and *qkr54B* mRNAs, which encode two of the RBPs we surveyed, are themselves HOT RNAs. Additionally, we note that the set of HOT RNAs, as well as RIP-seq targets in general, spans a wide range of gene expression levels (see Supplemental Figure 2) and is not biased toward highly expressed RNAs.

**Figure 2.**
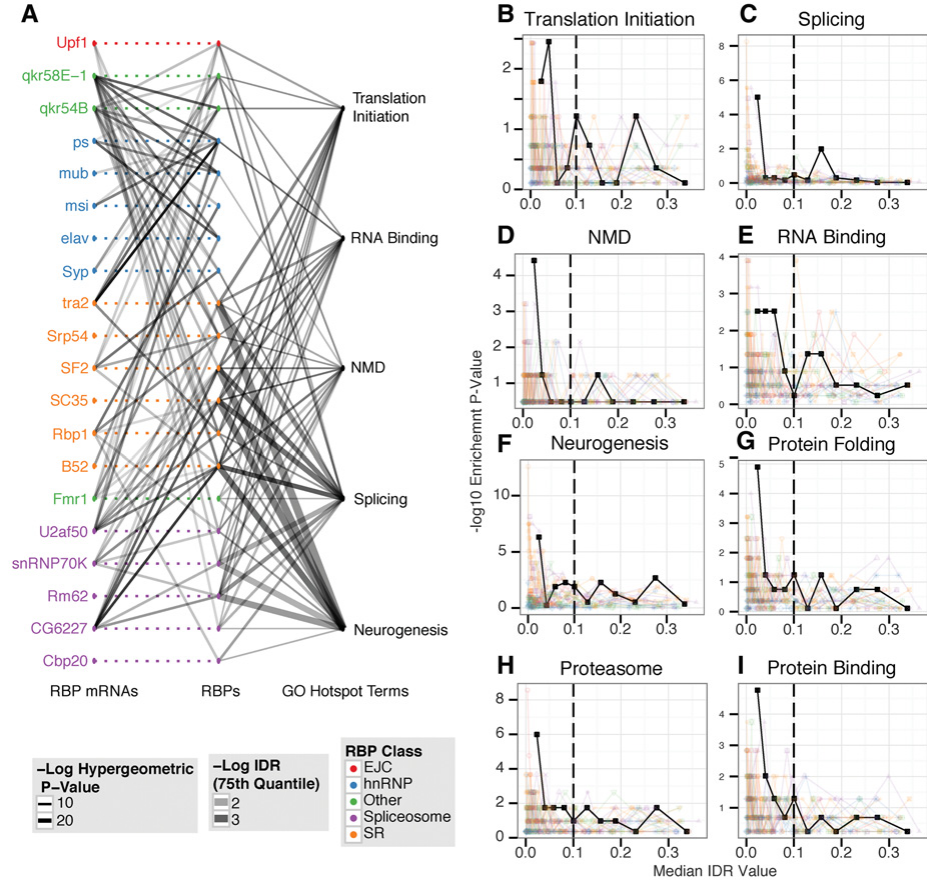
RBP-RNA–binding network. (*A*) This plot presents a global view of the RNA–protein interaction network. Each point in the *center* column represents an RBP (RIP-seq experiment). Corresponding points on the *left* represent each RBP's mRNA. Dashed lines represent hypothetical binding events that cannot be observed due to the overexpressed background. Lines join an RBP and an RBP's mRNA if significant binding is observed (Methods), and the lines are shaded according to the strength of binding (−log IDR value) for this interaction. Points on the *right* represent the set of genes annotated with the corresponding hotspot GO term. Lines are drawn between an RBP and a hotspot GO term if the bound set of RNAs significantly overlaps (*P* < 0.01) the GO term set. The thickness of these lines represents the significance of the overlap between the corresponding sets of RNAs. The shading of these lines indicates the binding strength of this set of bound RNAs (defined as the 75th percentile of the –log IDR values for the bound RNAs). (*B*–*I*) HOT RNAs are driven by the most enriched RNAs. Each plot represents the enrichment for a single hotspot GO term gene set across all experiments. The one solid line represents the median IDR value for each RNA for of all RBPs, and each transparent line represents a single RBP. Each point represents 100 RNAs binned by IDR value in increasing order. The *y*-value for each point represents the −log hypergeometric *P*-value for the overlap between the 100 bound RNAs and the GO term gene set. Each plot represents the “down-the-rank-list” enrichment for a particular hotspot GO term: (*B*) translation initiation, (*C*) splicing, (*D*) NMD, (*E*) RNA binding, (*F*) neurogenesis, (*G*) protein folding, (*H*) proteasome, and (*I*) protein binding.

A number of biological process GO terms are strongly enriched in the HOT RNAs (Supplemental Table 9), with the strongest being nuclear mRNA splicing (GO:0000398, adjusted *P* < 0.001), neurogenesis (GO:0022008, adjusted *P* < 0.001), and NMD (GO:0000184, adjusted *P* < 0.05). We also observe enrichment for the molecular function GO terms RNA binding (GO:0003723, adjusted *P* < 0.05) and translation initiation (GO:0003743, adjusted *P* < 0.05). When we rank the target RNAs by their local IDR, using this score as a proxy for a direct measure of binding affinity or fractional occupancy, we find that the most strongly associated RNAs drive the enrichment of the HOT RNA enriched GO terms (Fig. 2). Collectively, HOT RNAs include mRNAs of almost a quarter of the genes involved in RNAi (five out of 22, including *Dcr-2* and *AGO2*) and almost half (five out of 12) of the genes involved in NMD, despite consisting of only 3% of expressed genes (Fig. 2). The hnRNP and quaking-related RBPs contribute much less significantly to HOT RNA GO term enrichments (rank rum *P* < 0.0005). However, at least one hnRNP or quaking-related protein targets 92% of HOT RNAs and thus contributes strongly overall to the biological GO term enrichments of HOT RNAs.

### Binding events identified by RIP-seq are functional

To assess potential biological functions of the RNA–protein interactions identified in this study, we compared our RIP-seq results to RNA-seq data generated by Brooks et al. (2015) after RNAi knockdown of 14 of the RBPs included in this study (SRP54, CG6227, RM62, MUB, QKR54B, UPF1, B52, RBP1, ELAV, SNRNP70K, SYP, SC35, TRA2, and FMR1). Although the extent of protein depletion was not monitored by Brooks et al. (2015) due to the lack of antibodies, depletion of the target mRNAs was confirmed by RT-PCR and analysis of the RNA-seq data (see Supplementary Materials of Brooks et al. 2015). By using splicing changes calculated with JuncBase by Brooks et al. (2015), we observed statistically significant overlaps (max *P* < 0.05) between the splicing events altered upon RNAi knockdown of an RBP and the RIP-seq targets for the same RBP (Table 1). The overlap between RIP-seq targets and affected splicing events for TRA1 and FMR1 fell below statistical significance (max *P* < 0.05). It is possible that the proteins were not sufficiently depleted to affect the splicing of many of the targets of TRA2 and FMR1. However this may also be reflective of biology as FMR1 is known to play a role in mRNA localization and has not been reported to directly regulate splicing. Similarly, although it is known that TRA2 regulates splicing, it is also possible that TRA2 has additional functions and that many of its interaction targets are not splicing regulatory targets. Nonetheless, we conclude that strong overlaps for the vast majority of tested RBPs provide statistical evidence for the functional importance of the interactions identified by RIP-seq (Fisher combined *P* < 10^−100^).

**Table 1.**
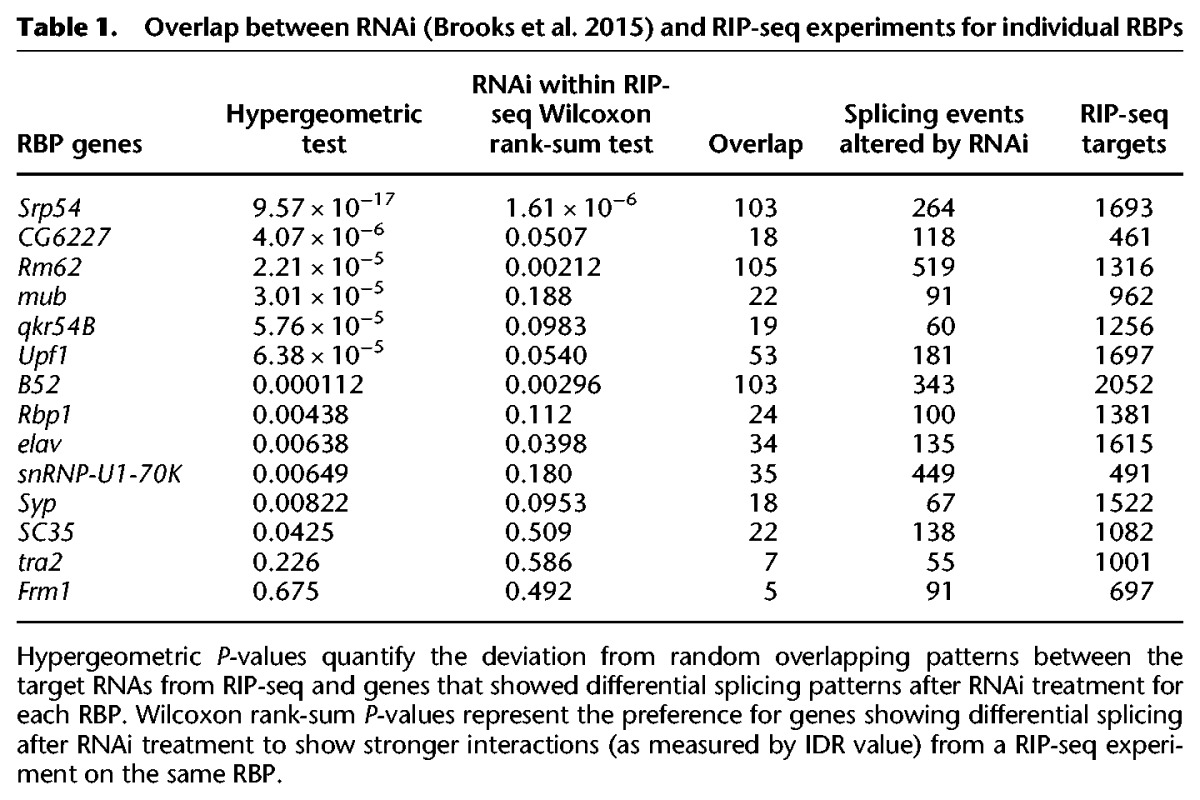
Overlap between RNAi (Brooks et al. 2015) and RIP-seq experiments for individual RBPs

### RNP complexes contain proteins and their encoding mRNAs

**Figure 3.**
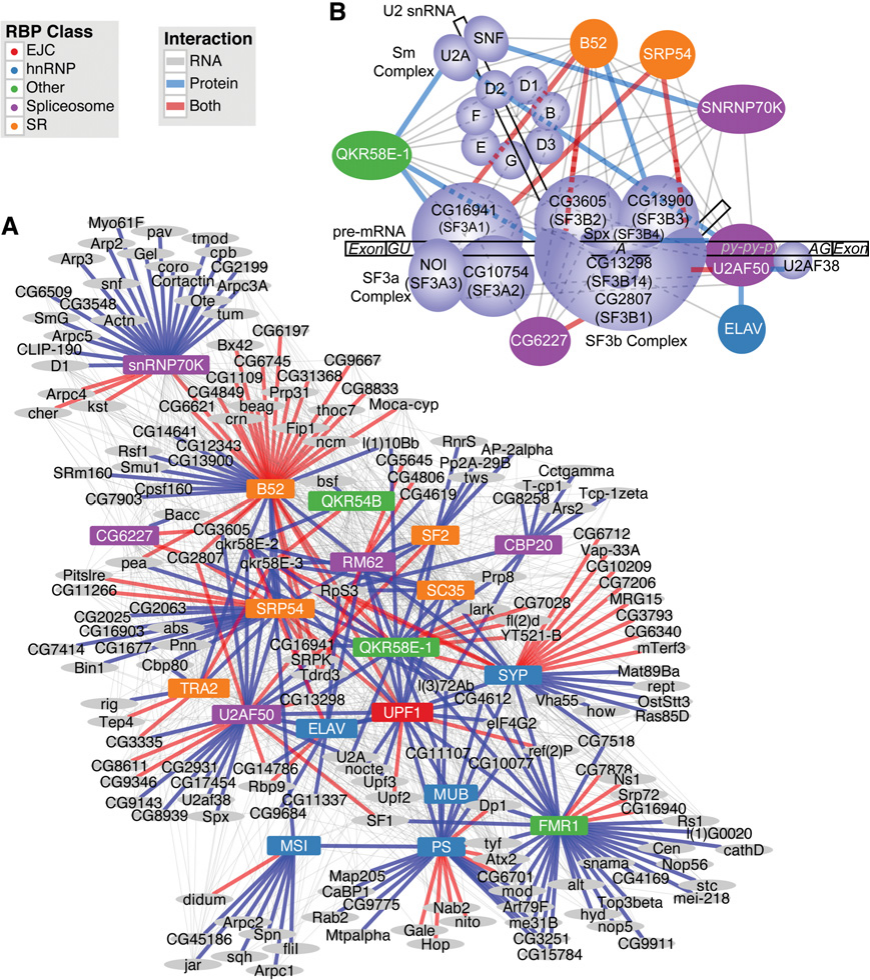
RBP–protein–RNA interactions. (*A*) Plot represents combined interactions between RBPs and all proteins pulled down in at least one experiment, as well as their corresponding transcripts. Edges are drawn where an RBP participates in an interaction with a gene. Gray lines indicate RBP–RNA interactions; blue lines, RBP–protein interactions; and red lines, both interactions with the same gene. (*B*) Diagram of the U2 snRNP (adapted from Kotake et al. 2007), showing the interactions between core proteins of the U2 snRNP and the RBPs from this study. Only those RBPs involved in protein–protein interactions are presented. The U2 snRNP is composed of U2 snRNA, the SF3a and SF3b splicing complexes, and the Sm proteins. Lavender-colored proteins are components of the U2 snRNP (along with U2AF50). RBPs from this study are colored according to their primary class designation used consistently throughout the article. Lines indicate the type of interaction as in *A*.

As mass spectrometry was conducted on each of the IP fractions from which RNA was eluted for sequencing, we can identify the proteins associated with all RBP baits. We obtained at least one confident interacting protein for all but one (RBP1) of the 20 RBPs. We observed an average of 13 proteins associated with each RBP and a total of 198 proteins coassociated with at least one RBP. We confirmed protein–protein interactions for the pairs SC35:QKR58E-2, QKR58E-1:LARK, and U2AF50:UPF1 using reciprocal co-IP experiments with an alternative tag (Supplemental Fig. 3). These simultaneously validate the targeted interactions and our protein tagging strategy (Supplemental Methods). We also compared our results to a database of published interactions (www.droidb.org), and found that 44.6% have been previously reported (greater than 40-fold enrichment, parametric permutation test, *P* < 10^−16^). The coassociated proteins are strongly enriched for mRNA-binding (GO:0003729, *P* < 10^−13^) molecular function despite removing many interactions between RBPs from this study due to possible cross-contamination (Supplemental Table 10). The associating proteins enriched for several terms are also enriched in the HOT RNAs, including both biological process and cellular component splicing-related terms. We observed highly significant overlap (hypergeometric *P* < 0.01) between the protein and the corresponding RNA targets for three (B52, SYP, and CG6227) of the 20 RBPs (Supplemental Table 11) and note that there is a strong tendency among all RBPs to cobind proteins and their mRNAs (Fisher's method *P* < 10^−8^). For example, the B52 protein interacts with the protein and mRNA products of *CG4849*. Hence, RNP complex members interact with the RNAs encoding interacting proteins. This indicates that post-transcriptional regulation is highly interconnected, hierarchical, and cross-regulatory, operating at both the transcript and protein levels (Fig. 3A).

We find significant enrichment for genes encoding components of the U2 snRNP and related proteins (GO:0005686, adjusted *P* < 10^−7^). Within this complex, we observe coordinated binding, where RBPs coimmunoprecipitate with proteins and the corresponding mRNAs that encode them (Fig. 3B), suggesting tight post-transcriptional control of the U2 snRNP complex by constituent and other RBPs. For example, we observe that four RBPs from this study (U2AF50, B52, SRP54, and CG6227) interact with both the RNA and protein expressed from *CG2807*, which encodes the ortholog of SF3B1 (SAP155), an integral component of the U2 snRNP complex. Furthermore, QKR58E-1 interacts with the CG2807 protein, and SNRNP70K interacts with the *CG2807* RNA*. CG16941*, which encodes the SF3A1 subunit of U2 snRNP, is another hub of interactions with protein–protein interactions with QKR58E-1; RNA–protein interactions with SNRNP70K and CG6227, the ortholog of yeast Prp5; and both protein and RNA interactions with the SRP54, U2AF50, and B52 proteins. Finally, we observe that B52 appears to play a central role as it interacts with many U2 snRNP components, including the RNAs of four Sm proteins—D1, D2, D3, and F—and three proteins—CG16941, CG13900 and CG2807—as mentioned above. Together these results are consistent with an intricate network of cross-regulatory interactions that control expression of the U2 snRNP components.

In addition to the experiments performed for this study, we investigated relationships to protein complexes and pathways reported in the DPiM, a protein–protein interaction map generated in the same cell line (Guruharsha et al. 2011). The DPiM contains 10 protein complexes containing 12 of the 20 RBPs in this study. For these RBPs, we observe associations with RNAs encoding proteins within the reported complexes for seven out of eight (not including two gene complexes) (Supplemental Table 11). These interactions include SNRNP70K within the DPiM complex 30 (DC30), where we find SNRNP70K binds RNAs encoding three of the seven proteins that compose this complex. DC482 is a complex containing only PS and MSI, which we confirmed, and we observed a strong association between PS and *msi* RNA but no significant evidence for the reciprocal interaction between MSI and *ps* RNA. DC52 includes QKR54B and SYP, which is replicated in our experiments (using SYP as bait). This complex includes six other proteins, four of which contain RNA-binding motifs (CG4612, CG7903, NITO, and QKR58E-3) and a fifth that contains an RNA helicase domain and has been implicated in RNAi (CG6701). QKR54B and SYP, as well as QKR58E-1, associate with the transcripts encoding QKR58E-3 and CG6701. Additionally, we see that QKR58E-1 strongly associates with this complex through both protein and RNA interactions (Supplemental Fig. 4). We find reciprocal RNA binding between the pairs of SYP and QKR58E-1, as well as QKR54B and QKR58E-1. Seven gene products interact with two of these three RBPs, and four genes interact with all three.

### hnRNP/QKRs associate with unique target RNAs and RNAs from ultracomplex genes

Recently, a small subset of genes was identified that each generates more than 100 mRNAs via complex alternative splicing, promoter use, and polyadenylation and are referred to as “ultracomplex genes” (UCGs) (Boley et al. 2014; Brown et al. 2014). Most, but not all, UCGs are expressed principally in neural tissue (Brown et al. 2014). UCGs are rare in the *Drosophila* transcriptome; 255 are expressed in S2R+ cells. Nonetheless, we find that UCGs are enriched among the RNA targets of RBPs. UCGs are 25% more likely to be associated with at least one RBP than would be expected at random (binomial *P* < 10^−38^), and this enrichment is driven largely by hnRNP/QKRs (rank sum *P* < 0.0005) (Supplemental Table 12). Mice bearing mutations in the orthologs of *qkr54B* and *qkr58E-1* exhibit neural developmental phenotypes (Sidman et al. 1964). Our results show that the targets of QKR54B and QKR58E-1 are not enriched for genes involved in neurogenesis. However, QKR58E-1 shows among the strongest enrichment for UCGs (hypergeometric *P* < 5 × 10^−10^).

In addition to enrichment for UCGs, hnRNP/QKRs tend to associate with unique target RNAs that other RBPs from this study do not target (rank sum *P* < 0.001) (Supplemental Table 12). The most striking examples are ELAV and MSI that have 26% and 19% of their RNA targets associated with no other RBP studied. Additionally, RNAs with low expression (RPKM < 1 in control samples) are 1.7-fold more likely to show binding to hnRNP/QKR (binomial *P* < 10^−54^). In particular, *Ccn* RNA, which encodes a growth factor implicated in neurogenesis, is detected at very low levels in the control samples (0.27 RPKM) and 19 of the IP samples (max of 0.72 RPKM) yet is enriched more than 7000-fold by SYP (592 RPKM). This indicates a highly specific and strong association between SYP and this neurogenesis-related mRNA.

### hnRNP/QKRs associate with extended 3′ UTRs

**Figure 4.**
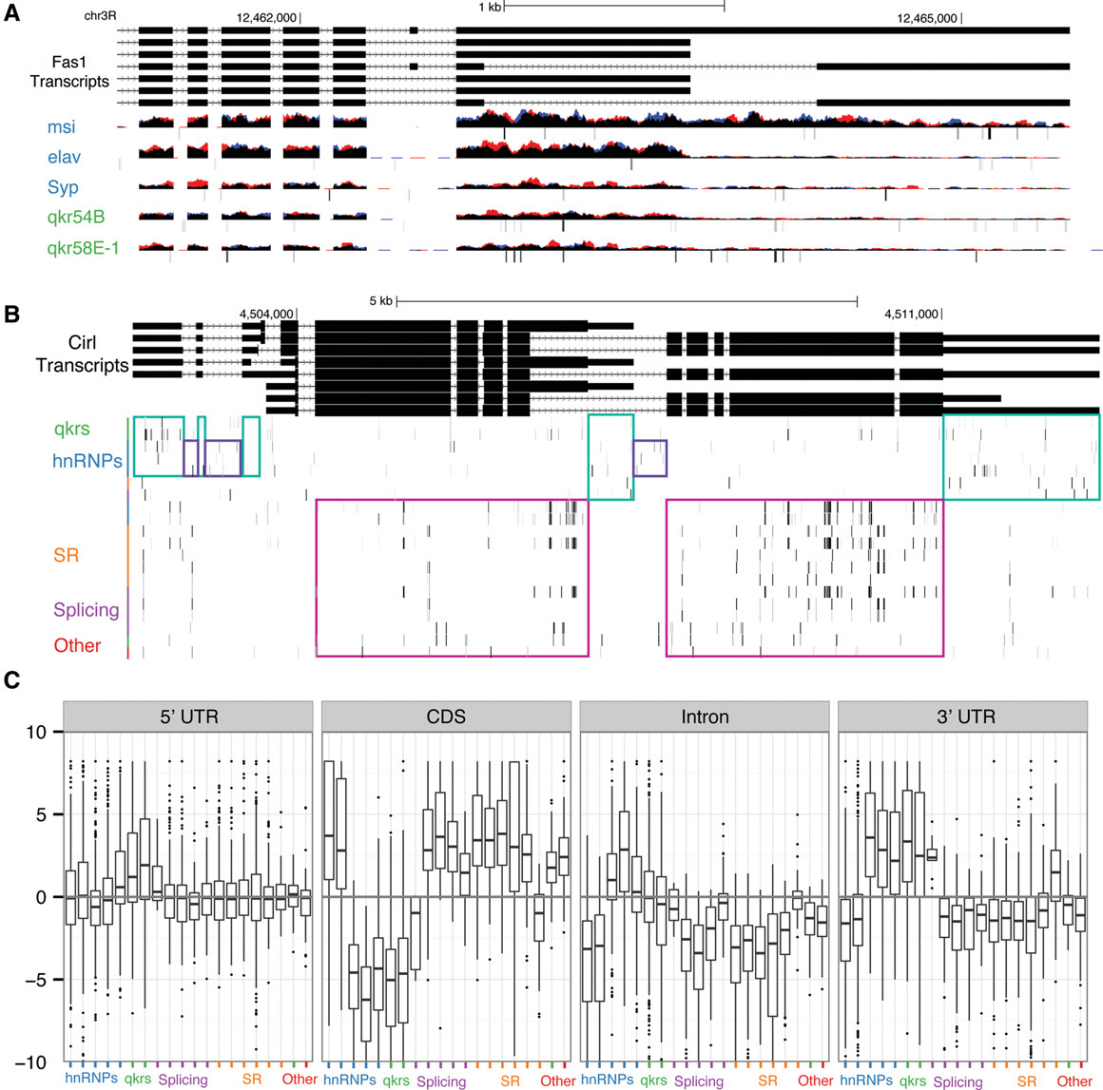
Gene structure binding. (*A*) The 3′ UTR region of the *Fas1* locus, where we observe MSI binding specifically to the 3′ UTR extended isoform. It has been previously reported that this 3′ UTR extension is controlled by ELAV. (*B*) *Cirl* is a hotspot RNA in our analysis (bound by, in order of lowest to highest IDR value, SRP54, U2AF50, B52, RBP1, RM62, CG6227, MUB, TRA2, QKR58E-1, PS, and SNRNP70K). We note that motifs hits cluster in the gene structure regions. (*C*) This plot represents the enrichment of each RBP's motif along gene structure (5′ UTR, CDS, 3′ UTR). The annotation was collapsed into regions that are only observed as a particular gene structure. Significant motif *k*-mers (top 1% most likely *k*-mers given the RBP PWM) are then identified across the transcriptome and overlapped with the gene structure. Each point represents the enrichment of motif-hit proportion within a gene element over the length of the gene structure element at the locus. Note that only enriched loci for each RBP with at least 20 motif hits are plotted.

It has been previously published (Hilgers et al. 2012) that *elav* is necessary and sufficient to produce several 3′ UTR extensions in *Drosophila* and that this action is dependent on the direct association of ELAV with target transcripts. We investigated the associations between the RBPs and RNAs expressed from 363 genes with previously reported 3′ UTR extensions that are expressed in S2R+ cells (Smibert et al. 2012). We find that ELAV associates with 34% of these RNAs containing 3′ UTR extensions (*P* < 10^−15^). However, several other hnRNP/QKRs are also strongly associated with RNAs containing 3′ UTR extensions (QKR54B 30%, *P* < 10^−16^; QKR58E-1 36%, *P* < 10^−21^; MSI 26%, *P* < 10^−18^). We find that MSI associates with 52 RNAs containing 3′ UTR extensions that are not detectably associated with ELAV. We manually reviewed each of the eight ELAV targets reported by Hilgers et al. (2012) and found equal or stronger association to 3′ UTR extended isoforms by QKR54B, QKR58E-1, and MSI than with ELAV (Supplemental Fig. 5). One striking example is *Fas1*, where MSI associates with isoforms, including the extended 3′ UTR, and ELAV associates only with the shorter isoforms (Fig. 4A). These results indicate that several hnRNP/QKRs in addition to ELAV associate with neural-specific 3′ UTR extensions. These proteins potentially play roles in either poly(A) site selection, RNA localization, RNA stability, or translation regulation of the 3′ UTR extended isoforms.

### Gene region motif enrichment

We next sought to identify sequence motifs enriched in the RNA targets associated with each RBP. However, since the approach we used enriches for full transcripts (Supplemental Fig. 6) rather than small, RBP-protected fragments, identification of sequence motifs must be performed by considering the entire sequence of the all possible RNAs at each enriched locus. Extant methods are not available to determine sequence specificity given a set of bound loci within a complex transcriptome, where many genes encode multiple transcripts. We developed a method that identifies enriched sequence signatures within a set of RNAs compared with all expressed genes and, if statistically significant sequence signatures are found, combines these to produce a sequence motif for each RBP (Methods). We identified motifs for each factor. The RBP encoded by *pasilla* (*ps*) interacts specifically with repeats of YCAY (Brooks et al. 2011), a motif we recover (Supplemental Fig. 7). Additionally, we compared our motifs to those discovered using the in vitro RNAcompete method (Ray et al. 2013) and found strong correspondence (Supplemental Methods).

The motif enrichment across target mRNA gene structure suggests that motif analysis may provide insight into the regions of mRNAs bound by particular RBPs. For instance, analysis of the gene *Cirl* (a HOT RNA) reveals a consistent pattern of UTR binding for some factors and of CDS binding for others (Fig. 4B). We asked if any RBPs’ motif showed preferential binding in the 5′ UTR, CDS, introns, and/or 3′ UTR and computed the enrichment of motifs across the transcript body (Methods). In general, the hnRNP/QKRs tend to bind UTRs more than the spliceosome or SR proteins do (Fig. 4C), though we observe that two hnRNPs, PS and MUB, show enrichment in CDS regions, while a motif of SR protein SRP54 is enriched in the 5′ UTR. ELAV targets are strongly enriched for genes with alternative 3′ UTRs, as expected, but the strongest enrichment is observed for MSI, which shows statistically significant enrichment in over three quarters of genes in *Drosophila*. We also found significant enrichment within the 5′ UTR for QKR54B, QKR58E-1, and SYP. Splicing factors (excluding CBP20) and SR proteins show a mean 2.7 enrichment *z*-score (*P* < 0.01) for motif hits in CDS regions. The EJC release factor UPF1 shows strong binding to the CDS, consistent with its role in NMD that detects splicing irregularities in coding sequence.

### Noncoding RNA–RBP interactions

We sequenced total RNA without a size fractionation step, thus recovering unpolyadenylated noncoding RNA targets, which include microRNAs (miRNAs), snRNAs, small nucleolar RNAs (snoRNAs), and scaRNAs, as well as unpolyadenylated long noncoding RNAs. We visually inspected many examples of these targeted RNAs and discovered that the majority of targets are due to the enrichment of the RNA precursors (i.e., retained introns containing noncoding RNAs). However, we also observed several examples of significant enrichment for “intergenic” noncoding RNAs (e.g., *snRNA:U5:14B*, *snRNA:U2:14B*, and *snRNA:U2:34ABb*). For example, RBP1, MUB, and MSI all significantly enrich for the *snRNA:7SK* RNA; in fact, MSI enriches for this RNA over 19-fold. Intriguingly, ELAV displays a very strong (590-fold) interaction with *snRNA:U5:35D*, and QKR58E-1 enriches *RNaseP:RNA* over sevenfold. In addition, there are 236 annotated noncoding RNAs (e.g., *CR31044*) that interact with between one and 11 RBPs: For example, *CR31044*, which encodes a ∼5-kbp RNA that contains miR-279 and miR-996, interacts with 11 RBPs, the strongest of which is SYP. Similarly, 10 RBPs interact with *CR43651*, which encodes a ∼1-kbp RNA hosting miR-14; the strongest interactor in this case is PS with a 65-fold enrichment. These results identify RBPs that may participate in the biogenesis of specific miRNAs.

We also find that 10 RBPs target one or more small functional RNAs, and in total, 19 small functional RNAs are targeted by at least one RBP (Supplemental Tables 3, 4). These include six of 144 expressed snoRNAs, four of nine miRNAs, seven of 18 snRNAs, and two of 14 scaRNAs. Of the 10 RBPs, ELAV targets include the most: eight small functional RNAs. No other RBP targets more than four. As mentioned earlier, two U1 snRNAs, *snRNA:U1:82Eb* and *snRNA:U1:95Cc*, interact with SNRNP70K, consistent with its known role in the U1 snRNP complex (Mount et al. 1983), and are among the most enriched RNAs in any IP in this study (84-fold and 81-fold, respectively).

### Enriched intronic regions

**Figure 5.**
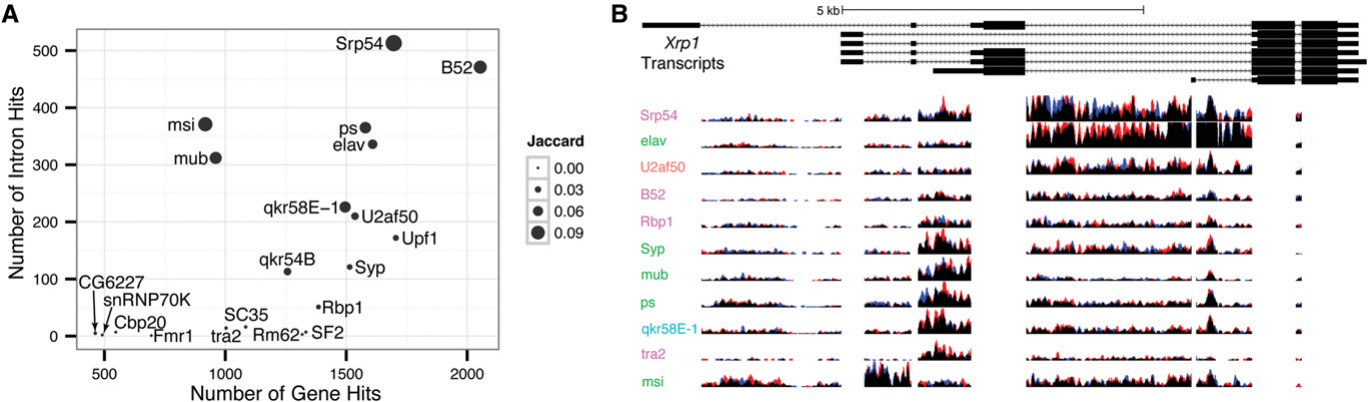
Retained intron signal in the data. (*A*) The number of intron and gene level targets is represented for each RBP on the *y*- and *x*-axes, respectively. The amount of overlap between each RBP's intron and gene level targets, as measured by the Jaccard index at the locus level, is indicated by each point's size. (*B*) The *Xrp1* locus is indicative of several genes that produce different cohorts of RBPs binding to different retained introns. The exon regions of reads are removed from this figure. The height of each sequence track is 20 bases per kilobase per million mapped reads. Red and blue portions of the tracks indicate biological replicates.

In addition to investigating the enrichment of particular mRNA transcripts at the gene level, we also queried introns for evidence of enrichment (Methods). We find that while gene level enrichments correlate well (ρ = 0.62) with intron enrichment loci across RBPs, there are several factors with many gene level targets that do not show a similar signature at the intron level, consistent with intron targeting as a feature of some RBPs and not others (Fig. 5A). Two factors in particular, B52 and SRP54, are enriched for retained introns at almost twice as many loci as any other RBP (Supplemental Table 13).

We find individual introns are targets of multiple factors. We also find gene loci with multiple introns that are targets of distinct cohorts of factors. A striking example is the *Xrp1* locus, which encodes a DNA-binding protein and is a HOT RNA (targeted by 17 RBPs) (Fig. 5B). *Xrp1* contains seven introns, five portions of which do not overlap other annotated features and, hence, are amenable to differential expression analysis (Methods). We find differential intronic enrichment for four of these introns. For example, the second intron is strongly targeted by MSI, but not any other factors, while the fourth intron is preferentially targeted by SRP54 and ELAV. Several other loci with marked differential intron retention include *MRP* and *crol*, as well as at the loci of two of the RBPs in this study, *ps* and *Syp*.

## Discussion

We obtained genome-wide RNA–protein and protein–protein interaction profiles for 20 RBPs. The combined use of next-generation sequencing and mass spectrometry on a single immunoprecipitate for each RBP provided new insights into the composition of ribonucleoprotein complexes in metazoans. Validation of RNA–protein interactions was performed by comparison to an RNA sequencing data set generated from RNAi-depleted cells (Brooks et al. 2015) demonstrated the functional importance of these complexes in splicing regulation.

We found that strongly bound RNAs included HOT RNAs that interact with most of the factors in our study. These included many of the genes encoding proteins in the RNAi and NMD pathways, those related to neurogenesis, other RNA-binding and splicing factors, and components of the proteasome. This is consistent with previous reports (Nogueira and Springer 2000; Keene 2007) that genes involved in post-transcriptional regulation tend to be regulated post-transcriptionally. Feedback loops are a central idea in cellular biology, and it is striking that feedback appears to function broadly at the level of an entire regulatory process. Integrative analysis of RBP protein and mRNA interaction profiles revealed ubiquitous interactions with mRNA and protein products of the same gene. Furthermore, we find that RBPs that participate in the same protein complex tend to reciprocally bind the mRNAs of their interaction partners. A striking example of this was presented for the RBPs that interact with the protein components of the U2 snRNP and the RNAs encoding them. Hence, we find that widespread post-transcriptional regulation of post-transcriptional regulators may be an emergent property of local cross-regulation, where RBPs of a complex tend to regulate their interaction partners. Similar patterns have been observed among transcription factors acting in the same pathway, for example, global cross-regulation within the gap gene network (Jaeger 2011). We find that protein interaction–associated post-transcriptional regulation is common and, hence, constitutes a general layer of feedback in the hierarchy of gene regulation.

The hnRNP/QKR proteins bound a more diverse repertoire of target RNAs than other classes of RBP. We found that hnRNP/QKRs in general were strongly associated with UCGs, genes with many promoters, alternative splicing events, and/or polyadenylation sites. In contrast, SR proteins bound largely overlapping sets of post-transcriptional regulators, with few targets bound by only a single member of this class. This is consistent with their known biochemical redundancy (Shepard and Hertel 2009). We find that the hnRNP *Syp* mRNA is itself a target of QKR58E-1 and, reciprocally, that SYP binds mRNAs of *qkr58E-1*, and we detected protein–protein interactions between SYP and QKR58E-1. The mRNAs of the quaking-related factor *held out wings* (*how*) are targets of both quaking-related factors we surveyed, and HOW is a protein interaction partner of SYP. Overall, we find extensive coregulation and interaction among UCGs and the RBPs that target them.

We found that splicing factors and the EJC component UPF1 tend to bind CDSs of target mRNAs, while hnRNP/QKRs are enriched in UTRs. While several factors, including ELAV, are strongly enriched in 3′ UTRs, we found that other hnRNP/QKRs, particularly MSI, show even stronger association with 3′ UTR extensions. It was previously reported that ELAV is both necessary and sufficient for these extensions to exist at eight genes (Hilgers et al. 2012), but global binding patterns indicate that MSI interacts with the extended 3′ UTRs and may play an important role in some aspect of their biology. Thus, ELAV may modulate the biogenesis of extended 3′ UTRs, while MSI binds to the extended UTRs and regulates the translation, localization, or stability of the mRNAs.

Our data are consistent with the colocalization of mRNAs of RBPs and the proteins they encode. Furthermore, these associations between interacting proteins and mRNA products from the same genes could be ribosome proximal or ribosome mediated. It could be that the protein complexes studied here undergo cotranslational assembly. This is also an intriguing possibility. Importantly, our assays measure time and space averages across ensembles of homogeneous, but not identical or synchronized cells. Hence, while it is clear that the proteins copurify and bind the same RNA targets, it may be that these associations occur on different individual RNA molecules that are neither spatially nor temporally localized with the proteins they encode. Additional assays, particularly high-content imaging approaches, will be needed to resolve these possibilities and to elucidate the intriguing biology at the basis of feedback in post-transcriptional regulatory networks.

## Methods

### Clone construction and transfection

Full-length ORFs for each RBP were obtained from an expression clone set generated for the DPiM project (Guruharsha et al. 2011). These ORFs were recombined into an expression vector along with a C-terminal Flag-HA tag (for clone construct, see Supplemental Fig. 8). Competent cells were transformed with the clones and incubated overnight. Each clone was -sequenced at the 5′ end to check for target mismatches. Plasmid DNA was purified and transiently transfected into two replicate *Drosophila* S2R+ cell cultures. Expression of the tagged protein was induced for a 24-h period before harvesting cells. For details, see Supplemental Methods within the sections Construction of the Flag-HA Expression Clone Set and Transfection and Recombinant Expression of Bait Proteins in Cell Culture.

### Protein isolation and processing

Cell lysate was clarified and put through a single-step anti-HA purification step. Unbound proteins were removed with extensive washes, and then bound complexes were released by competition. Of each sample, 30% was processed for LC/MS/MS, 60% was used for RNA purification, and 10% was used for quality controls. For details, see Supplemental Methods within the sections Protein Isolation and Liquid Chromatography-Mass Spectrometry (LC/MS/MS).

### Sequencing/mapping

RNA sequencing libraries were prepared using the Illumina mRNA sample preparation kits as described by the manufacturer, but both the poly(A) selection and RNA fragmentation steps were omitted. Libraries were sequenced on an Illumina HiSeq 2000 to generate single-end 50-bp reads. Reads were mapped to the *Drosophila* genome using TopHat (Trapnell et al. 2009) as previously described (Graveley et al. 2011). For details, see Supplemental Methods within the sections RNA Preparation and Sequencing/Mapping.

### Identification of differentially bound RNAs

After filtering and sequence based validation, differentially bound RNAs were identified in each biological replicate using the DESeq R package (Anders and Huber 2010). IDR values were computed with *P*-values, from biological replicates, using the IDR R package (Li et al. 2011), developed as part of the ENCODE Project Consortium as a measure of biologically reproducible findings. We required target RNAs to have a 10% local IDR value or lower in order to confirm biological reproducibility and at least a 50% increase in expression to ensure potential biologically effect. For details, see Supplemental Methods within the section Identification of Differentially Bound RNAs.

### GO term enrichment

Reported GO term enrichments were calculated using the hypergeometric distribution for the gene set of interest compared with all expressed genes. For details, see Supplemental Methods within the section GO Term Enrichment.

### RBP motif discovery and gene region motif enrichment

Novel RNA-binding sequence motif preferences were discovered as follows. At each gene, the set of unique 7-mer locations across all transcripts is obtained. The genomic sequence of each 7-mer is recorded. The enrichment of each 7-mer sequence is determined for the target genes of an RBP compared with the set of nontarget, expressed genes using the hypergeometric *P*-value. The most enriched 7-mers produce a single motif using the MEME algorithm (Bailey and Elkan 1995) with transformed enrichment *P*-values as 7-mer weights.

Gene region motif enrichments are calculated by identifying the presence of *k*-mer matches to the top 0.1% of sequences corresponding to the discovered motif. For each gene locus, a gene region enrichment *z*-score is calculated using the binomial test for the fraction of motif hits to a gene region against the null of the fraction of sequence within that gene region. For details, see Supplemental Methods within the sections Global RBP Binding Profile Comparison, RBP Motif Discovery, and Gene Region Motif Enrichment.

## Data access

All mapped reads data from this study have been submitted to the NCBI Gene Expression Omnibus (GEO; http://www.ncbi.nlm.nih.gov/geo/) under accession number GSE37756.

## Supplementary Material

Supplemental Material
