# Supplementary material for: Extensive cross-regulation of post-transcriptional regulatory networks in Drosophila
Source: Genome Res. 2015 Nov;25(11):1692–702. doi: 10.1101/gr.182675.114 (PMC4617965; doi:10.1101/gr.182675.114)
Supplement: Supplemental Material [file supp_25_11_1692__index.html]

Extensive cross-regulation of post-transcriptional regulatory networks in Drosophila — Extensive cross-regulation of post-transcriptional regulatory networks in Drosophila — Supplemental Material 

# Extensive cross-regulation of post-transcriptional regulatory networks in *Drosophila*

## Supplemental Material

**Files in this Data Supplement:**

- Supp Figures.pdf
- Supp Analysis scripts.zip
- Supp Table 1.xlsx
- Supp Table 2.xlsx
- Supp Table 3.xlsx
- Supp Table 4.xlsx
- Supp Table 5.xlsx
- Supp Table 6.xlsx
- Supp Table 7.xlsx
- Supp Table 8.xlsx
- Supp Text.docx
- Supp Table 9.xlsx
- Supp Table 10.xlsx
- Supp Table 11.xlsx
- Supp Table 12.xlsx
- Supp Table 13.xlsx
