# Supplementary material for: Extensive cross-regulation of post-transcriptional regulatory networks in Drosophila
Source: Genome Res. 2015 Nov;25(11):1692–702. doi: 10.1101/gr.182675.114 (PMC4617965; doi:10.1101/gr.182675.114)
Supplement: Supplemental Material [file supp_gr.182675.114_SuppText.docx]

**Supplementary Results**

**RBP functional groups bind the mRNAs of functionally related proteins**

The co-association of two or more RBPs with a single target RNA occurs broadly throughout the transcriptome. Pairwise intersections provide a natural similarity (and, conversely, dissimilarity) measure between any two RBPs (Methods). Multi-dimensional scaling (MDS), a generalization of Principle Component Analysis, is a powerful technique for visualizing the relationships between data points in high natural dimension (Borg and Groenen 2005). MDS of the co-associations into two dimensions (Supp. Figure 9a) reveals that RBPs from related functional groups (e.g. the SR proteins) bind overlapping sets of target RNAs. This clustering becomes tighter for the three major functional groups when, rather than considering the overlaps in the sets of associated RNAs, the similarity of annotated GO term enrichment profiles (across all biological GO terms) is examined (Methods). As several analyses indicate a strong relationship between the hnRNP class of RBPs and the two quaking related proteins (QKR54B and QKR58E-1), we will refer to these proteins collectively as hnRNP/QKRs.

MDS using GO term profiles indicates that functionally related RBPs associate with mRNAs encoding functionally related proteins (Supp. Figure 9b). Clusters are tighter under the GO similarity measure than raw transcript overlap (Supp. Figure 10). We observe the following ratios between mean within-group distances and mean between-group distances: SR proteins (transcript overlap 0.85, functional overlap 0.69); Spliceosome (transcript overlap 0.98, functional overlap 0.92); hnRNP/QKR (transcript overlap 0.95, functional overlap 0.92). However, these cluster measurement differences do not rise to the level of statistically significant. Both the hnRNP/QKR and SR protein classes functional clustering is driven in part by the HOT RNA enriched term neurogenesis, as defined by the GO terms that provide the largest decreases of within-group distances when removed from the analysis (Methods). Additionally, SR proteins cluster due to mitotic spindle organization (GO:0007052) and translation (GO:0006412) while splicing-related RBPs functionally cluster primarily due to the HOT RNA enriched term splicing as well as telomere capping (GO:0016233).

**Comparison of RIP-seq and RNACompete Derived Motifs**

Position specific score matrixes (PSSMs) have been determined by RNAcompete, which measures the binding specificity of purified recombinant proteins with a pool of randomized RNA, for 50 *Drosophila* RBPs (Ray et al. 2009), of which 13 are included in our study. To determine the extent to which RNAcompete PSSMs are sufficient to explain interaction strength in S2R+ cells (quantified by negative log IDR value), we examined the predictive power (Methods) of the RNAcompete motifs, and compared these to motifs derived from our data. We found that in all cases the RNAcompete motif alone was not sufficient to predict RIP-seq binding patterns (Supp. Figure 10). Hence, as has been found in numerous studies of transcription factors (Harrison et al. 2011) (Kaplan et al. 2011), the interactions between RBPs and their target RNAs cannot be strongly predicted by simply using PSSMs scores derived from *in vitro* biochemical assays. We find that the predictive power of the motifs derived from our RIP-Seq experiments, which had no access to quantitative information about strength of binding, is greater for 10 out of 13 RBPs than the RNAcompete motifs, with an average of 35% better fold-change in predictive power (Supp. Figure 11).

**RIP-seq Derived Motifs Cluster within Functional Groups**

We quantified the similarity of PSSM binding models for our factors using Kullback-Leibler divergence (Aerts et al. 2003). MDS analysis reveals relationships between classes of factors (Supp. Figure 12). We note that the spliceosome components and SR proteins show the most similar RIP-seq derived motifs, driven primarily by a strong “AGG” submotif. This is in contrast to the RNAcompete motifs, which show significant differences within these classes (Supp. Figures 7, 12, 13). We note as well that for U2AF50 in particular, the motif discovered in our experiments diverges significantly from the RNAcompete motif. In the case of U2AF50, the differences between our motif and the RNAcompete motif may be partly due to the fact that we used only exon sequences in our motif discovery. In addition, U2AF50 is known to form a tight heterodimer with U2AF38, and interacts with other proteins, which could impact the binding specificity of U2AF50 in our RIP-seq experiments. Since the RIP-seq derived motifs correspond only to enriched sequence signatures, and may not reflect the direct binding specificity of the factors, the few factors for which the RIP-seq and RNAcompete motifs strongly differ may be due to the detection of sequences associated with other RNA binding proteins or co-factors.

**Co-Immunoprecipitation of TAP-tagged and FLAG-HA-tagged proteins**

The availability of TAP-tagged versions of some of the proteins in the FLAG-HA-tagged RBP panel allowed us to test pairs of proteins that we had identified as interacting, to see whether the same interactions could be recovered when one member of each pair was expressed with a different C-terminal tag. These pairs were: SC35-TAP:qkr58E-2-FLAG-HA, SC35-TAP:qkr58E-3-FLAG-HA, qkr58E-1-TAP:lark-FLAG-HA, U2af50-TAP:Upf1-FLAG-HA, and U2af50-TAP:elav-FLAG-HA. An empty vector construct (containing only the FLAG-HA tag) was used as a negative control, and a pair of known interacting proteasome proteins were used as a positive control, prosbeta3-TAP:Pros28.1-FLAG-HA. Western blots of whole cell lysates and purified protein complexes eluted from IgG resin (which binds the Protein A moieties of the TAP tag) showed that all the tagged proteins were expressed, and three of the RBP:RBP interactions were recovered (SC35:qkr58E-2, qkr58E-1:lark, and U2af50-TAP:Upf1) while the other two were not (Supplementary Figure 3).

**Supplementary Methods**

**Construction of the FLAG-HA expression clone set**

Full-length Open Reading Frames (ORFs) for each RNA-binding protein were obtained from a clone set generated for the DPiM project (Guruharsha et al. 2011). ORFs from the BDGP Gold Clone Collection were transferred from the Donor Vector to the [pMK33-C-TAP-FLAG-HA-BD](http://flybase.org/reports/FBmc0003027.html) expression vector using Cre recombinase (see <http://www.fruitfly.org/about/methods/cre_recombinase.html> for details). This vector contains a metallothionein (Mt) promoter for induction using copper, splice donor (SD/HIS) and splice acceptor (SA) sites, and harbors the open reading frame (ORF) from the transcript being studied starting with its ATG initiation codon. *In vivo* splicing of the primary transcript juxtaposes the ORF in-frame with the sequence encoding the FLAG-HA tag (YKDDDDKVKLYPYDVPDYAAA) and a stop codon. The *Drosophila* actin *5C* terminator sequence provides signals for the polymerase to stop transcription. The plasmid also contains ampicillin (Amp^r^), and hygromycin (hygromycin^r^) resistance genes, as well as a chloramphenicol-resistance cassette (promoter and Cm^r^ gene) that is removed from the transcript by the splicing event. The vector is one of a series of tag-containing expression vectors based on pMK33 (Veraksa et al. 2005; Yu et al. 2011).

We transferred ORFs from the BDGP *Drosophila* melanogaster expression-ready clone set to the pMK33-C-FLAG-HA Acceptor Vector for expression of FLAG-HA fusion proteins (Yu et al. 2011). For recombination reactions, 200 ng of expression-ready Donor clone and pMK33-C-FLAG-HA Acceptor Vector were recombined in a final volume of 10 μl for 15 minutes at 25°C in a thermal cycler in the presence of *Cre* recombinase (0.2 units) and recombinase buffer supplemented with BSA (0.1 mg/ml) (Clontech #631614) according to Clontech manual PT3460-1. *Cre* recombinase was inactivated by incubating the reaction at 70°C for 10 minutes. From this reaction, 5 μl was mixed with 50 μl of chemically competent TAM-1 cells in a 96-well plate format (Active Motif #11096). Following incubation on ice for 30 minutes, the competent cells were heat-shocked for 30 sec in a 42°C water bath. SOC media, 150 μl, was added to the transformed cells and allowed to incubate at 37°C for 1 hour with shaking at 225 rpm. The entire incubation mixture was plated onto LB-agar plates containing 50 μg/ml Chloramphenicol and 7% sucrose (w/v) to select for the correct recombinant plasmid. After overnight incubation at 37°C, a single colony was inoculated into a well of a 96-well, 2 ml, square-well, round-bottom plate (E&K Scientific #662000) filled with 1.2 ml 2XYT media supplemented with 50 μg/ml Chloramphenicol and allowed to grow overnight at 37°C with shaking at 300 rpm. Each clone was sequenced at the 5’ end to check for target mismatches. For sequence verification, DNA was prepared by direct heat lysis of a 5 μl aliquot of an overnight culture in a 96-well PCR plate (E&K Scientific #489096). DNA from the lysed bacterial cells was sequenced using Big Dye Terminator v3.1 ready reaction mix (Applied Biosystems #4337457) and the sequencing primer: 5'-GCCAATGTGCATCAGTTGTGGTC-3'. Sequencing samples were analyzed on a conventional capillary electrophoresis instrument (e.g., ABI 3730/3730xl DNA Analyzer). Glycerol stocks were generated and stored for each isolate. A collection of 9,889 C-terminal FLAG-HA epitope-tagged clones representing 8,779 unique genes was thus generated. Clones can be obtained from the Drosophila Genome Research Center: <https://dgrc.cgb.indiana.edu/vectors/store/infusion.html>.

The “TAP-tagged” clones used in the co-immunoprecipitation validation experiments were generated as above but using the [pMK33-C-TAP-BD](http://flybase.org/reports/FBmc0003027.html) expression vector (with a tag based on that of Puig, et al. "The tandem affinity purification (TAP) method: a general procedure of protein complex purification." (Puig et al. 2001), and three ORFs, SC35, qkr58E-1, U2af50, and prosbeta3, from the BDGP *Drosophila* *melanogaster* expression-ready clone set.

**Transfection and Recombinant Expression of Bait Proteins in Cell Culture**

Plasmid DNA was prepared using the PureLink™ HQ Mini Plasmid Purification Kit [Invitrogen K2100-01], and eluted with 50ul sterile water into a 1.5ml DNA LoBind microfuge tube (Eppendorf). The DNAs were quantified and assayed for A_260_/A_280_ ratio >1.8 using a NanoDrop spectrophotometer (ThermoFisher). Drosophila S2R+ cells were grown at 25°C in non-tissue culture treated polystyrene flasks (Corning Incorporated).

Each clone was transiently transfected into two replicate 54ml cultures of Drosophila S2R+ cells (1 x 10^6^ cells per ml) grown in a T-150 flask in Schneider’s Drosophila Media (GIBCO 21720) with 10% heat-inactivated fetal bovine serum (GIBCO 26140079). Twelve micrograms of each DNA was combined with 300ul of Effectene (Qiagen) following the manufacturer’s protocol. Twenty-four hours after transfection, expression of the tagged protein was induced with 0.35mM CuSO_4_. This level of CuSO_4_ has been tested to induce a low-to-medium level of recombinant protein expression for a majority of representative clones. Twenty-four hours after induction, cells from each culture were harvested by blowing medium at the surface of the flask to dislodge the cells using a Pasteur pipet. The cells were washed thrice with 1x PBS at room temperature in a 50ml conical tube, and whole-cell lysates were prepared with 5ml/culture of Lysis Buffer (25 mM NaF, 1 mM Na_3_VO_4_, 50 mM Tris pH 7.5, 1.5 mM MgCl_2_, 125 mM NaCl, 0.2% IGEPAL, 5% glycerol + Complete™ Tablets, 1 per 50ml, 40U/ml RNasin (Promega) by trituration with a 10ml plastic serological pipet, followed by gentle rotation at 4°C for 30min. Transfection efficiency and the level of bait protein expression in each cell line was analyzed by direct immunofluorescence using fluorescein-conjugated anti-HA antibody (Roche Applied Science, Clone 3F10, cat.# 11988506001).

**Protein Isolation**

Prior to binding to affinity resin, lysates from cultures transfected with FLAG-HA-tagged bait proteins were quick-thawed, then clarified by passage through a 0.45 micron PVDF syringe filter (Millipore, Inc.). The entire process of purification is carried out at 4^o^C to preserve the integrity of protein complexes and prevent potential degradation. Cleared cell lysates were put through a single-step anti-HA purification using an immunoaffinity resin (Clone HA-7, Sigma-Aldrich A2095). Each tube of immunoaffinity resin along with captured proteins was transferred to AcroPrep 96 Filter Plates with 1.2 µm, Supor membranes (PALL #5065). Unbound proteins were removed by washing the resin 3 times with 800ul Lysis Buffer (composition as above, but containing 10U/ml RNasin) followed by four additional washes with 800ul PBS (GIBCO) to remove non-specific interactors and traces of detergent from previous washes. The bound complexes were released from the resin by competition, with 200ul of a solution of synthetic HA peptide YPYDVPDYA (250 µg/ml; Biosynthesis, Inc.) in PBS. Two successive elutions are carried out at 37^o^C and the eluates combined for further processing. Thirty percent of each sample was processed for LC/MS/MS analysis, sixty percent was subjected to RNA purification, and 10 percent was retained for analytical purposes. Each of these three portions was frozen at -80^o^C following the affinity purification.

Lysates from TAP-tagged cultures were lysed and processed as above, but bound to IgG-Sepharose 6 Fast Flow (GE Healthcare) that had been cross-linked with dimethyl pimelimidate (ThermoPierce). The resin-bound proteins were washed as above, then eluted with IgG Elution Buffer (Thermo Scientific). 20% of each sample was analyzed by electrophoresis and western blotting using a Novex apparatus, using 4-12% NuPAGE gels and 0.45um PVDF membranes (Life Technologies). Blots were probed with either HRP-rabbit anti-mouse IgG (Jackson Immunochemicals) or HRP rat anti-HA (Roche) and visualized with Supersignal West Dura substrate (ThermoPierce).

**Liquid Chromatography-Mass Spectrometry (LC/MS/MS)**

Mass spectrometric analyses of purified protein samples were performed at the Taplin Biological Mass Spectrometry Facility, Harvard Medical School. The sample from each protein purification was prepared for LC/MS by precipitating with cold 20% trichloroacetic acid (TCA). The resultant protein pellets are washed by vortexing 5 seconds and centrifuging at 14,000 x g for 30 minutes at 4^o^C, once with 10% TCA then four times with ice-cold acetone to remove free HA peptide present in the eluate; the pellets were then air-dried. The protein samples were subjected to in-solution overnight trypsin digestion (6.25 ng/ul in 50 mM Ammonium Bicarbonate). Tryptic peptides were analyzed by liquid chromatography MS/MS (LC/MS/MS). Peptides were separated across a 45 min gradient ranging from 10% to 35% (v/v) acetonitrile in 0.1% (v/v) formic acid in a (125 µm X 18 cm) C_18_ microcapillary column (Magic C18AQ, 5 µm particles, 200 Å pore size, Michrom Bioresources) and analyzed on-line on an LTQ XL™ (ThermoFisher, Inc.). For each cycle, one full MS scan was followed by ten MS/MS spectra on the linear ion trap XL from the ten most abundant ions. MS/MS spectra were searched using the SEQUEST algorithm against the *Drosophila melanogaster* predicted protein database (version 5.1). All peptide matches were filtered based on tryptic state, mass deviation, Xcorr and Dcorr. Unique peptides and total peptides identified for each protein were scored. The FLAG-HA tag sequence was added to the Drosophila predicted tryptic fragment database as a full set of C-terminal fusions. This allows identification of each C-terminal peptide from the bait proteins that contain a junction with the FLAG-HA sequence, and provides additional confirmation of a unique sequence for identification of true bait protein in any given sample. The false positive rate was calculated for each data set by determining the number of "reverse hits" using target-decoy database searches against a reverse-translated *Drosophila melanogaster* predicted proteome (Elias and Gygi 2007)**.**

**RNA Preparation**

The aliquot from each protein complex affinity purification that was to be used for RNA purification (60% of the total product, or 360ul) was thawed and extracted with 2.5 volumes of Trizol (USB) in a 2ml microfuge tube, vortexed, and after 5 minutes combined with 180ul chloroform, vortexed, and transferred to a Phase Lock Gel tube (5 Prime, cat. # 2302810), centrifuged 20 minutes at 14,000 x g, 4^o^C, and the upper aqueous phase was collected. The volume of the aqueous phase was measured with a pipettor, then added to an equal volume of 70% ethanol and applied to an RNeasy MinElute column (Qiagen cat. # 74204), and processed according to the manufacturer’s instructions, with a final elution in 12ul RNAse-free water (Ambion, AM9916). Each RNA was analyzed on an Agilent BioAnalyzer 1200 using a Total RNA Pico chip (Agilent 5067-1513).

**Sequencing/Mapping**

RNA sequencing libraries were prepared using the Illumina mRNA Sample Preparation kits as described by the manufacturer, but both the poly(A) selection and RNA fragmentation steps were omitted. Libraries were quantitated on an Agilent Bioanalyzer and sequenced on an Illumina HiSeq 2000 to generate single-end 50 bp reads. Reads were mapped to the Drosophila genome using tophat version 1.4.0 guided by the MDv1 annotation (Graveley et al. 2011) with the following settings: --no-novel-juncs, -a 6, -m 2, --min-intron-length 28, -I 200000, -F 0, -g 1, -x 60 and -n 2. Mapped reads are publicly available in the GEO database with accession number **GSE37756.**

**Control Filtering/Validation**

In order to obtain a confident set of control samples each empty vector control sample was tested for differentially enriched annotated RNAs versus all other empty control samples using the DESeq R package. One empty vector control sample produced a significant number (> 50 loci) of differentially enriched RNAs and was removed from further analysis, in particular from testing for differentially enriched RNAs in samples of interest. Similarly, each non-RBP sample was tested for differentially enrichment as compared to both the validated control samples as well as non-RBP samples. As each non-RBP experiment was conducted in biological replicate these replicates were tested together. None of the non-RBP samples produced more than a few (10) significantly bound (adjusted p-value < 0.05) RNAs and thus all (5 samples each in biological replicate) non-RBP samples were used for testing in the samples of interest.

**Sequence-based Transfection Validation**

In addition to western gel validation of RBP transfection the following sequence based method was applied to all samples. The raw sequence output of each experiment was queried for the exact FLAG-HA tag sequence immediately adjacent to the RBP of interest. A consensus sequence was created for each experiment by anchoring each read at the FLAG-HA tag sequence and recording the most frequent base at each position starting adjacent to the tag sequence. BLASTN (Altschul et al. 1990) was run on the consensus sequence against the drosophila “nr” database. All RBP samples of interest presented in this paper were confirmed via this method. Five non-RBP samples were used as negative controls as described in the previous methods section.

**Identification of Differentially Bound RNAs**

In order to confidently identify the differentially captured targets of each RBP of interest the following pipeline was implemented. Mapped reads were binned into gene counts with the htseq python package script htseq-count with setting intersection-strict against most current functionally characterized FlyBase annotation r5.57 (St Pierre et al. 2014). The DESeq tool (R version 3.0.2 and DESeq version 1.14.0) (Anders and Huber 2010) was used as the basis of the pipeline to identify differentially enriched RNAs. We note that differentially enriched RNAs have the same sequence signature as differential transcription in many extant studies, except that differential binding only results in more abundant transcripts. As such loci in which both replicates did not show a normalized fold change greater than one were filtered from downstream analysis. Also loci that did not show a sequencing depth greater than 1 read per kilobase per million mapped reads (RPKM) in the controls or any sample were removed from downstream analysis. We note that although some RNAs show low expression in the control samples we find significant evidence for strong binding at a select few loci (e.g. *Ccn*) and this do not use a strict RPKM cutoff from the controls alone. DESeq is thus an applicable tool in this setting after the appropriate filters are applied.

We note that all biochemical steps for the experiments in this dataset are identical aside from the RBP of interest’s RNA is transfected into the cells. Thus dispersion estimates at a particular locus are comparable across samples, up to sequence depth as required by the DESeq model. To take advantage of this replication schema gene level dispersion estimates were computed across all samples not being tested as well as controls at each locus for each RBP sample of interest (corresponding to the DESeq “per-condition” setting). Differential enrichment statistical significance values were then calculated on each biological replicate separately, thus producing two p-values for each RBP-RNA combination. Only RNAs that showed adjusted p-values less than one in both biological replicates as compared to the control samples were considered in further analysis.

In order to identify RNAs that are both strongly and reproducibly bound the IDR (R package; version 1.1.1) model (Li et al. 2011), a copula mixture model, was fit on the significance values (across all combinations of valid RBPs and RNAs). The IDR method has been extensively applied to assess reproducibility in biological experiments (Landt et al. 2012). Note that IDR cutoff values are quite reproducible when fit on each sample individually (Supp. Figure 14). Differentially enriched RNAs are defined as those that produce a local IDR value of less than 10% (corresponding to <10% chance of having resulted from the irreproducible component) as well as a minimal 50% increased fold change in both biological replicates.

**GO Term Enrichment**

In order to identify gene ontology (GO) terms that are identified within particular groups of genes we compute enrichment p-values for each GO term. The enrichment p-value is a hypergeometric p-value for the number of genes annotated with a particular GO term within a set of interest compared to all expressed genes (defined as in the previous section; at least one sample with greater than 1 RPKM). GO terms annotated to less than 5 genes are removed from analysis. All reported enrichment p-values are adjusted via the Benjamini, Hochberg (Benjamini et al. 2001) correction to control false discovery rate (FDR).

**Global RBP Binding Profile Comparison**

In order to visualize the binding partners, related characteristics of all RBPs as well as sequence binding preferences we applied dimension reduction techniques. The multi-dimensional scaling (MDS) algorithm (Cox and Cox 2001), which minimizes the difference between the input distance and the plotted two-dimensional representation, was applied to several different definitions of distance based on the bound RNAs of each RBP (using the R “cmdscale” function for metric MDS and “isoMDS” function for non-metric MDS that is part of the MASS package; version 7.3-33). Note that MDS output coordinates are only unique up to centering and dilation, thus coordinate values are omitted in plots.

The first distance is the Jaccard distance, one minus the size of the intersection divided by the size of the union, between the set of bound RNAs for any two RBPs. Note that non-metric MDS (which optimizes the rank of distances as opposed to the true distances) was applied to this distance definition since a reasonable fit could not be achieved with metric MDS. The second distance is defined on the vector of negative log (base 10) biological GO term enrichment values annotated to all bound RNAs. Note that values are capped at 10 to avoid outlier enrichment from driving distances. The “functional” distance between any two RBPs is defined as the cosine distance between the vectors of GO term enrichments for each RBP. The third distance is defined on the motifs discovered for each RBP as described in the next section as well as motifs discovered by the *in vivo* method RNAcompete method. In order to assess the distance between two motifs we used the Kullback Leibler divergence as has been done in previous studies with good success (Aerts et al. 2003). We define the distance by the smallest divergence between two motifs across all possible offsets requiring that at least four overlapping positions.

In order to compare relative clustering of RBP classifications we used the ratio of mean distances within a class to the mean distances between a class and all other RBPs. Thus lower ratios indicate tighter clusters (smaller distances within a class of RBPs than between that group and other RBPs). Note that these ratios are computed from the raw distance measures not the Euclidian distances from the MDS plots.

Specifically for the functional characterization we were interested in the GO terms that “drive” the clustering observed for a particular class. In order to identify those terms, we leave a single GO term out of the distance calculation and calculate the within class versus between class ratio. A positive differential with respect to the ratio including all terms indicates that a term causes a group to cluster more tightly. Those terms that are more prevalent outside a particular class of RBPs than within are removed, as these terms are not indicative of an attribute for that class of RBPs. These terms are driving the tighter clustering of a class by virtue of existing in all other RBPs. More prevalent is defined as a higher mean enrichment value within a class that in all other RBPs.

**RBP Motif Discovery**

Given that our differential enrichment analysis pipeline identifies loci, as opposed to linear sequences as in similar studies (Li et al. 2013) (Riordan et al. 2011), we have implemented the following pipeline in order to identify enriched motifs amongst the bound set of transcripts for each RBP in this study. We note that discovery of bound motifs in previous studies has proven particularly difficult and cannot be accurately obtained using existing software such as HOMER (Heinz et al. 2010) that takes linear, genomically disjoint sequences or MEME (Bailey and Elkan 1995) that in addition does not take into account enrichment, but only presence of a motif within a set of sequences.

The first step in the algorithm is to represent each locus as a vector of 7-mer counts. As the *Drosophila melanogaster* transcriptome contains a large number of alternative events (alternative transcript starts, stop as well as alternative splicing events) the set of 7-mers for each locus are defined as all unique genomic locations of 7-mers across all transcripts within each gene model. We note that we are not performing differential transcript isoform analysis that has proven to be a difficult problem to solve (Boley et al. 2014) and thus must take into account all annotated transcripts in our motif analysis. Note also that we are removing all global low complexity sequences from this analysis.

For each RBP the total count of each 7-mer across all bound loci is computed. Then the hypergeometric p-value for the enrichment of each 7-mer is calculated as compared to the background 7-mer totals amongst all expressed loci. We note that this distribution does not strictly follow the hypergeometric distribution as genes may contain multiple copies of a particular 7-mer, but note that randomly selected sets of genes produce reasonably uniform distribution of p-values under the hypergeometric model (Supp. Figure 15). The set of 7-mers that are significantly more enriched in the differentially enriched set of loci than one would expect at random are used to create the desired motif. Under the null model, of no sequence enrichment, p-values follow a uniform distribution. Thus we have that the minimal p-value follows a beta distribution with parameters alpha=1 and beta=1/# of 7-mers. We define a cutoff for a significant 7-mer as the one-sided 1% enrichment p-value of the beta distribution (6.13*10^-7^). We require that a bound set of interest produce greater than ten 7-mers which show significant enrichment (which all of our 20 RBPs do) in order to produce a motif. We only take the 50 most enriched 7-mers.

In order to produce a motif of variable width from the enriched set of 7-mers we generate a set of sequences with five copies of each 7-mer surrounded by ten bases of random sequence, thus approximately following the null distribution of the MEME model. This set of sequences is passed to the MEME algorithm (Bailey and Elkan 1995) along with weights corresponding to the transformed negative log enrichment p-values, thus forcing more enriched 7-mers to drive the motif signal. The transformation raises the minimum of the negative log p-value and 200 to the 0.75^th^ power and scales these values between 1/50^th^ and 1. The MEME algorithm produced a single motif per sample between 3 and 11 bases in width and is required to include each sequence in the produced motif (corresponding to the “oops” setting). The produced motif is taken as the experimentally discovered motif (Supp. Figure 7). A post-hoc filter was applied to trim the motif if less than 5% of the total information content lay in the outer-most positions of the motif.

**Gene Region Motif Enrichment**

Because the RIP-seq protocol does not include a cross-linking step we are able to observe only those transcripts that are differentially bound. In order to identify the gene region (i.e. un-translated region; UTR or coding sequence; CDS) preferences for each RBP we used the learned motif. First we identified the top 0.1% of hits (defined probabilistically by the position specific weight matrix) to the *Drosophila* transcriptome. We then intersected these locations with those regions that are only ever observed as a particular gene region (5’ UTR, CDS or 3’ UTR). Only genes in which each region type composed 2% of the total gene length were included. Also genes were required to have at least 20 hits for a particular motif to be included in order to remove genes with a small sample of motif locations. For these genes we compute a z-score by first computing the binomial test p-value, where the test statistic is the fraction of motif hits in a region type and the expected fraction is the sequence length for that region type over the total gene length. This p-value is transformed to a z-score by taking the inverse of the survival function for the Gaussian distribution.

**Statistical Procedures**

All statistical procedures were completed using the R software program (version 3.0.2). Rank sum p-values are computed using the one-sided “wilcox.test” function that is part of the stats package. Poisson-binomial p-values are computed using the “poibin” package, version 1.2 where the parameters are the fraction of expressed genes observed as differentially bound for each RBP.

**Supplementary References**

Aerts S, Van Loo P, Thijs G, Moreau Y, De Moor B. 2003. Computational detection of cis -regulatory modules. *Bioinformatics* **19 Suppl 2**: ii5-14.

Altschul SF, Gish W, Miller W, Myers EW, Lipman DJ. 1990. Basic local alignment search tool. *Journal of molecular biology* **215**: 403-410.

Anders S, Huber W. 2010. Differential expression analysis for sequence count data. *Genome biology* **11**: R106.

Bailey TL, Elkan C. 1995. The value of prior knowledge in discovering motifs with MEME. *Proceedings / International Conference on Intelligent Systems for Molecular Biology ; ISMB International Conference on Intelligent Systems for Molecular Biology* **3**: 21-29.

Benjamini Y, Drai D, Elmer G, Kafkafi N, Golani I. 2001. Controlling the false discovery rate in behavior genetics research. *Behavioural brain research* **125**: 279-284.

Boley N, Stoiber MH, Booth BW, Wan KH, Hoskins RA, Bickel PJ, Celniker SE, Brown JB. 2014. Genome-guided transcript assembly by integrative analysis of RNA sequence data. *Nature biotechnology* **32**: 341-346.

Borg I, Groenen PJF. 2005. *Modern multidimensional scaling : theory and applications*. Springer, New York.

Cox TF, Cox MAA. 2001. *Multidimensional scaling*. Chapman & Hall/CRC, Boca Raton.

Elias JE, Gygi SP. 2007. Target-decoy search strategy for increased confidence in large-scale protein identifications by mass spectrometry. *Nature methods* **4**: 207-214.

Graveley BR, Brooks AN, Carlson JW, Duff MO, Landolin JM, Yang L, Artieri CG, van Baren MJ, Boley N, Booth BW et al. 2011. The developmental transcriptome of Drosophila melanogaster. *Nature* **471**: 473-479.

Guruharsha KG, Rual JF, Zhai B, Mintseris J, Vaidya P, Vaidya N, Beekman C, Wong C, Rhee DY, Cenaj O et al. 2011. A protein complex network of Drosophila melanogaster. *Cell* **147**: 690-703.

Harrison MM, Li XY, Kaplan T, Botchan MR, Eisen MB. 2011. Zelda binding in the early Drosophila melanogaster embryo marks regions subsequently activated at the maternal-to-zygotic transition. *PLoS genetics* **7**: e1002266.

Heinz S, Benner C, Spann N, Bertolino E, Lin YC, Laslo P, Cheng JX, Murre C, Singh H, Glass CK. 2010. Simple combinations of lineage-determining transcription factors prime cis-regulatory elements required for macrophage and B cell identities. *Molecular cell* **38**: 576-589.

Kaplan T, Li XY, Sabo PJ, Thomas S, Stamatoyannopoulos JA, Biggin MD, Eisen MB. 2011. Quantitative models of the mechanisms that control genome-wide patterns of transcription factor binding during early Drosophila development. *PLoS genetics* **7**: e1001290.

Landt SG, Marinov GK, Kundaje A, Kheradpour P, Pauli F, Batzoglou S, Bernstein BE, Bickel P, Brown JB, Cayting P et al. 2012. ChIP-seq guidelines and practices of the ENCODE and modENCODE consortia. *Genome research* **22**: 1813-1831.

Li Q, Brown JB, Huang H, Bickel PJ. 2011. Measuring reproducibility of high-throughput experiments. doi:10.1214/11-AOAS466: 1752-1779.

Li Y, Zhao DY, Greenblatt JF, Zhang Z. 2013. RIPSeeker: a statistical package for identifying protein-associated transcripts from RIP-seq experiments. *Nucleic acids research* **41**: e94.

Puig O, Caspary F, Rigaut G, Rutz B, Bouveret E, Bragado-Nilsson E, Wilm M, Seraphin B. 2001. The tandem affinity purification (TAP) method: a general procedure of protein complex purification. *Methods* **24**: 218-229.

Ray D, Kazan H, Chan ET, Pena Castillo L, Chaudhry S, Talukder S, Blencowe BJ, Morris Q, Hughes TR. 2009. Rapid and systematic analysis of the RNA recognition specificities of RNA-binding proteins. *Nature biotechnology* **27**: 667-670.

Riordan DP, Herschlag D, Brown PO. 2011. Identification of RNA recognition elements in the Saccharomyces cerevisiae transcriptome. *Nucleic acids research* **39**: 1501-1509.

St Pierre SE, Ponting L, Stefancsik R, McQuilton P, FlyBase C. 2014. FlyBase 102--advanced approaches to interrogating FlyBase. *Nucleic acids research* **42**: D780-788.

Veraksa A, Bauer A, Artavanis-Tsakonas S. 2005. Analyzing protein complexes in Drosophila with tandem affinity purification-mass spectrometry. *Developmental dynamics : an official publication of the American Association of Anatomists* **232**: 827-834.

Yu C, Wan KH, Hammonds AS, Stapleton M, Carlson JW, Celniker SE. 2011. Development of expression-ready constructs for generation of proteomic libraries. *Methods in molecular biology* **723**: 257-272.
