# Supplementary material for: Extensive cross-regulation of post-transcriptional regulatory networks in Drosophila
Source: Genome Res. 2015 Nov;25(11):1692–702. doi: 10.1101/gr.182675.114 (PMC4617965; doi:10.1101/gr.182675.114)

Supplemental Figure 1

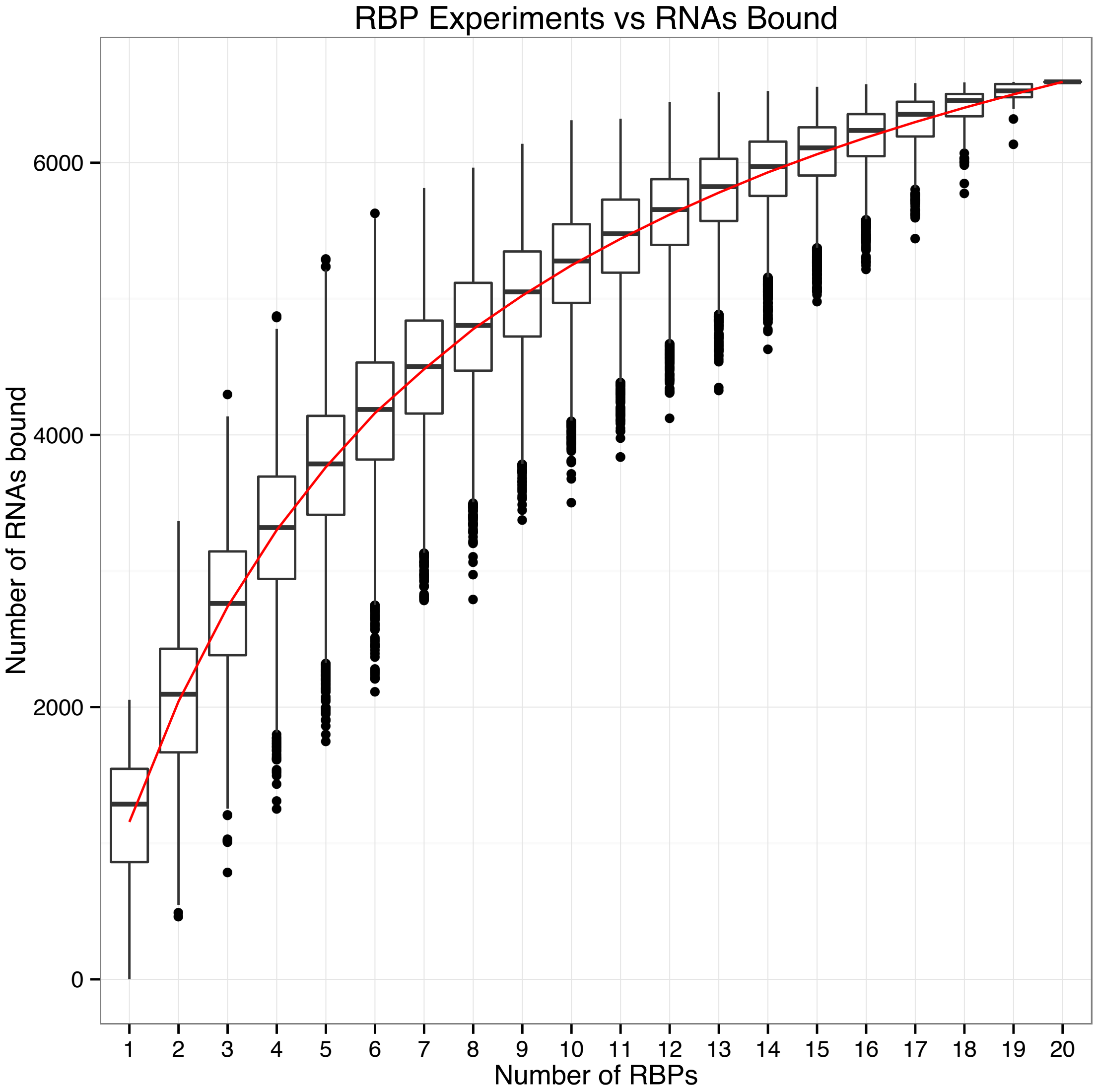

Supplemental Figure 2a

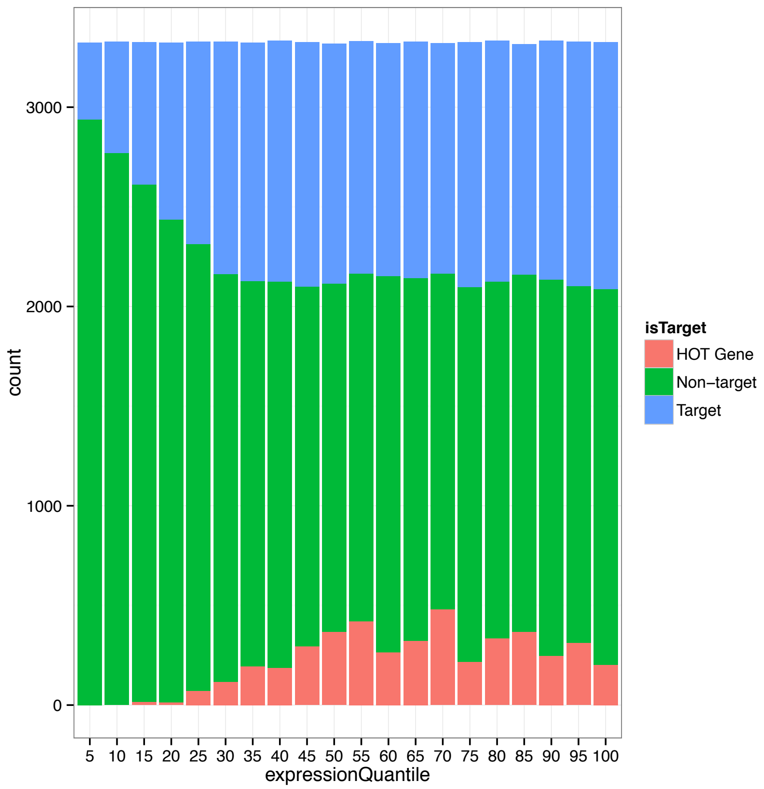

Supplemental Figure 2b

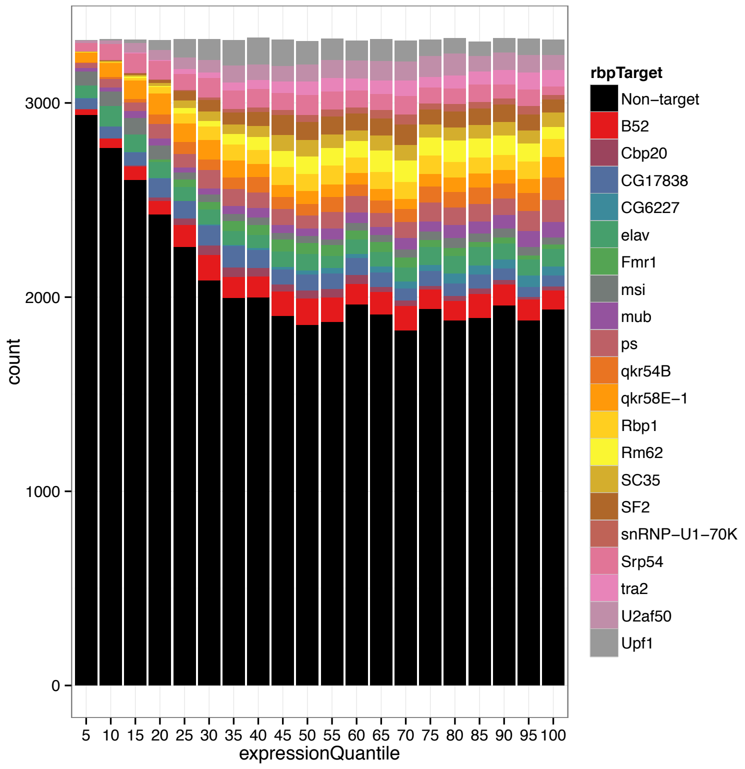

Supplemental Figure 3

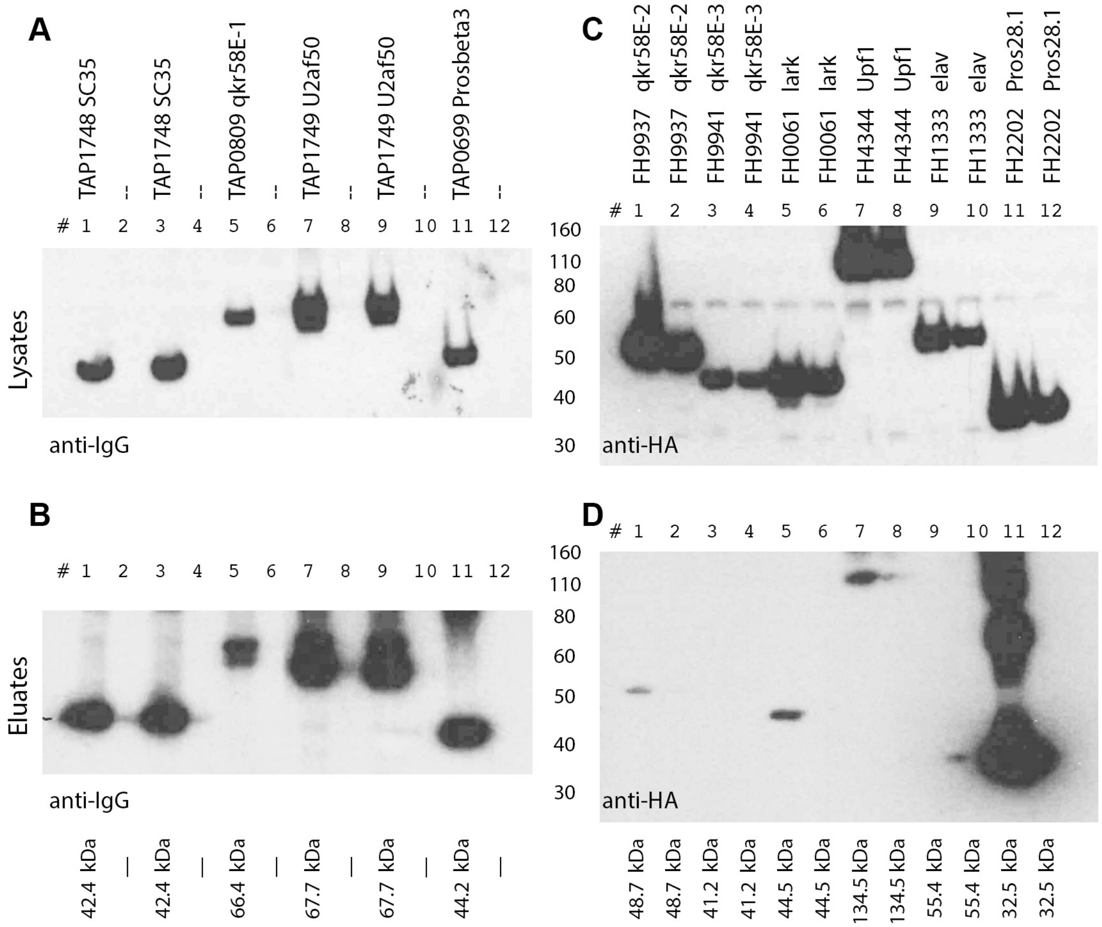

Supplemental Figure 4

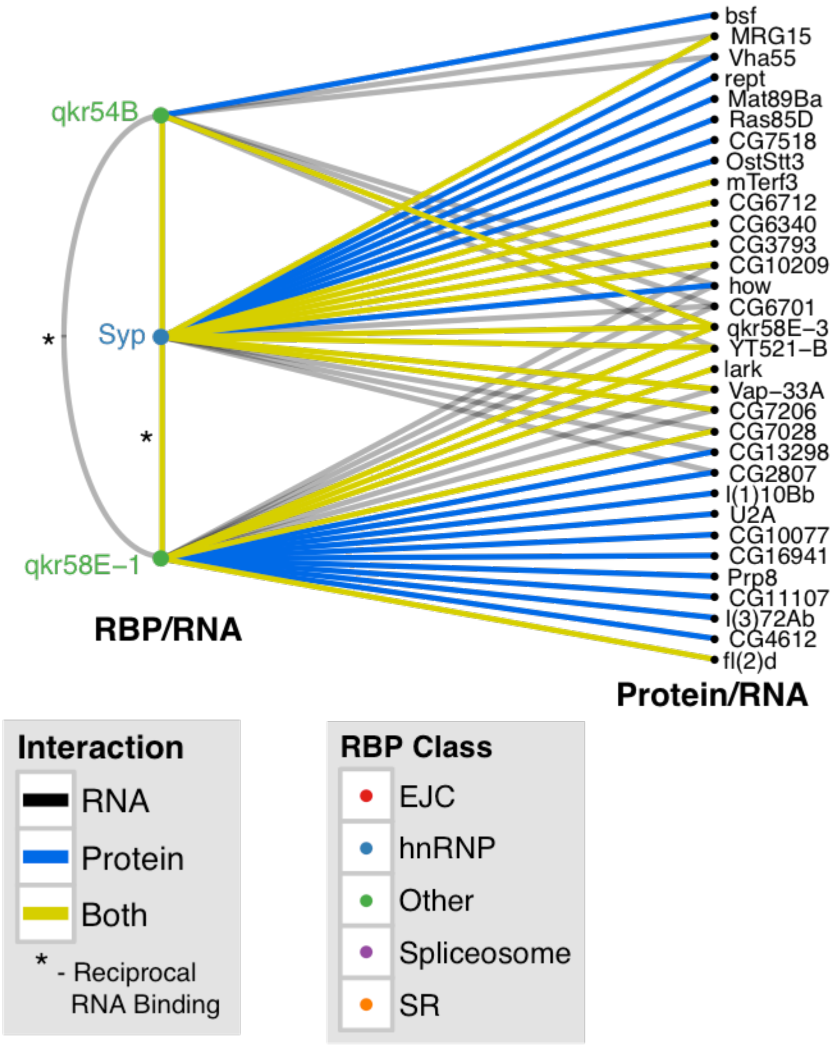

Supplemental Figure 5

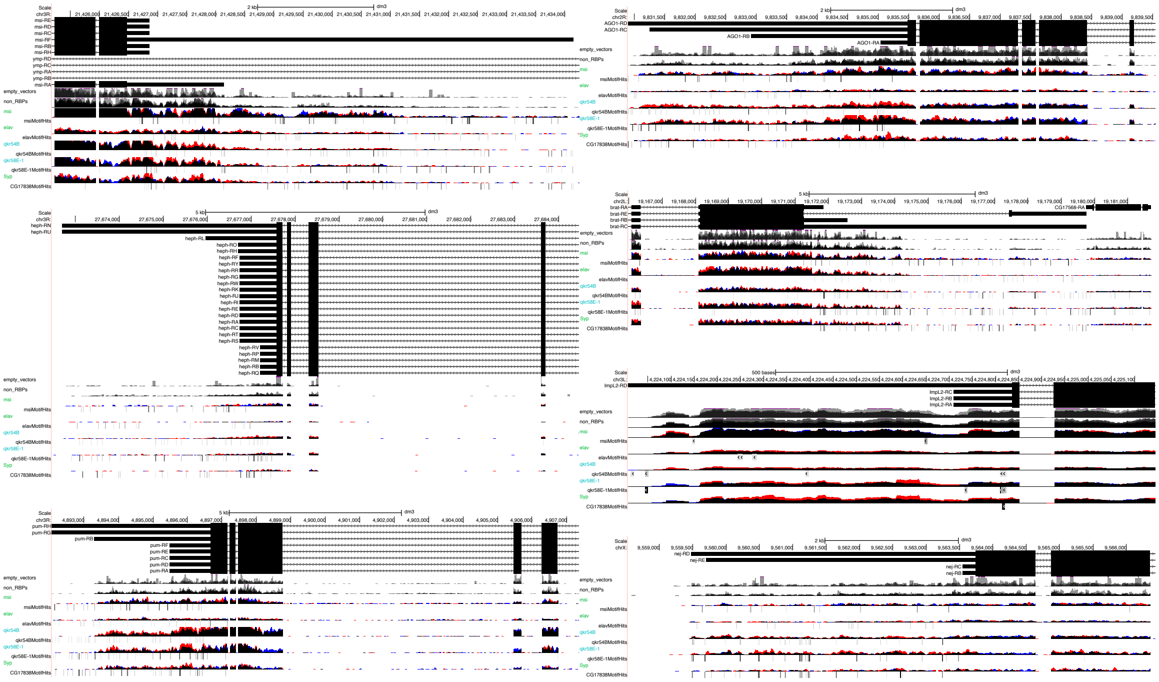

Supplemental Figure 6 (facetted by Motif Enrichment / Control Sample and all samples together)

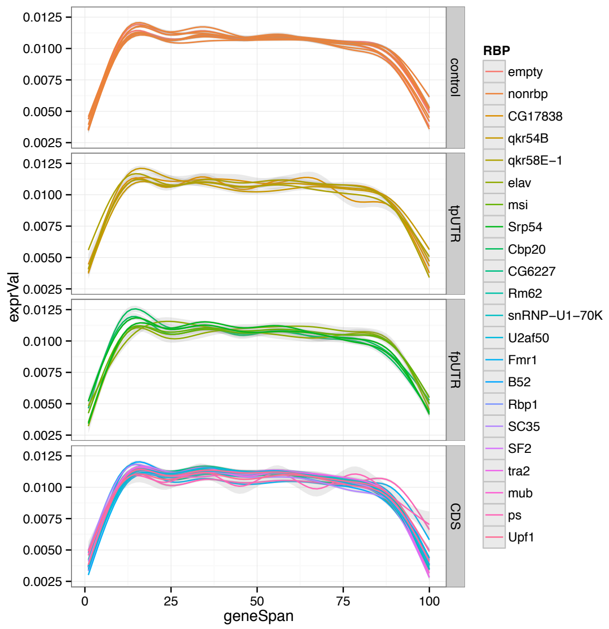

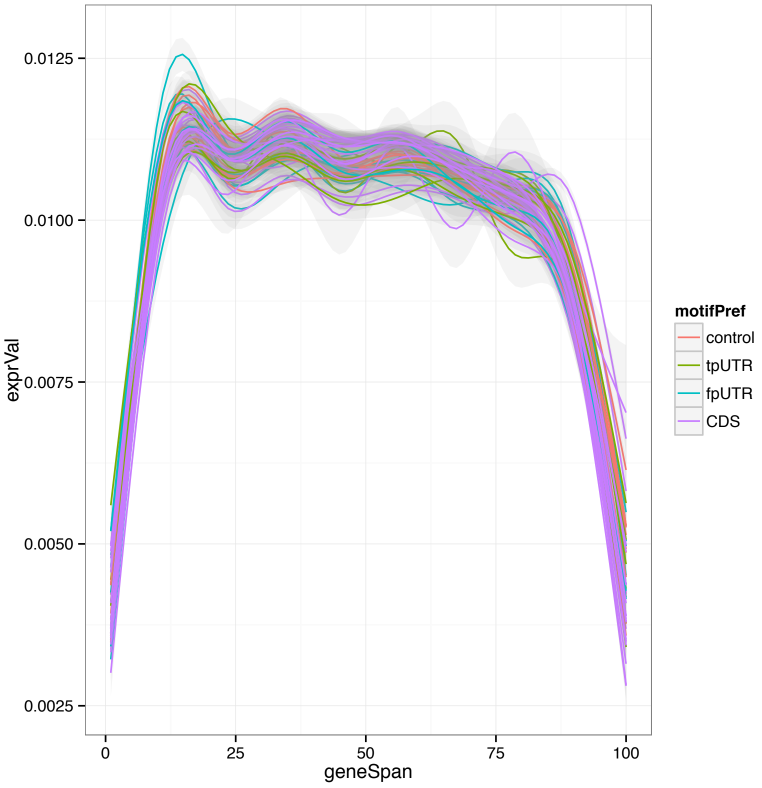

Supplemental Figure 7

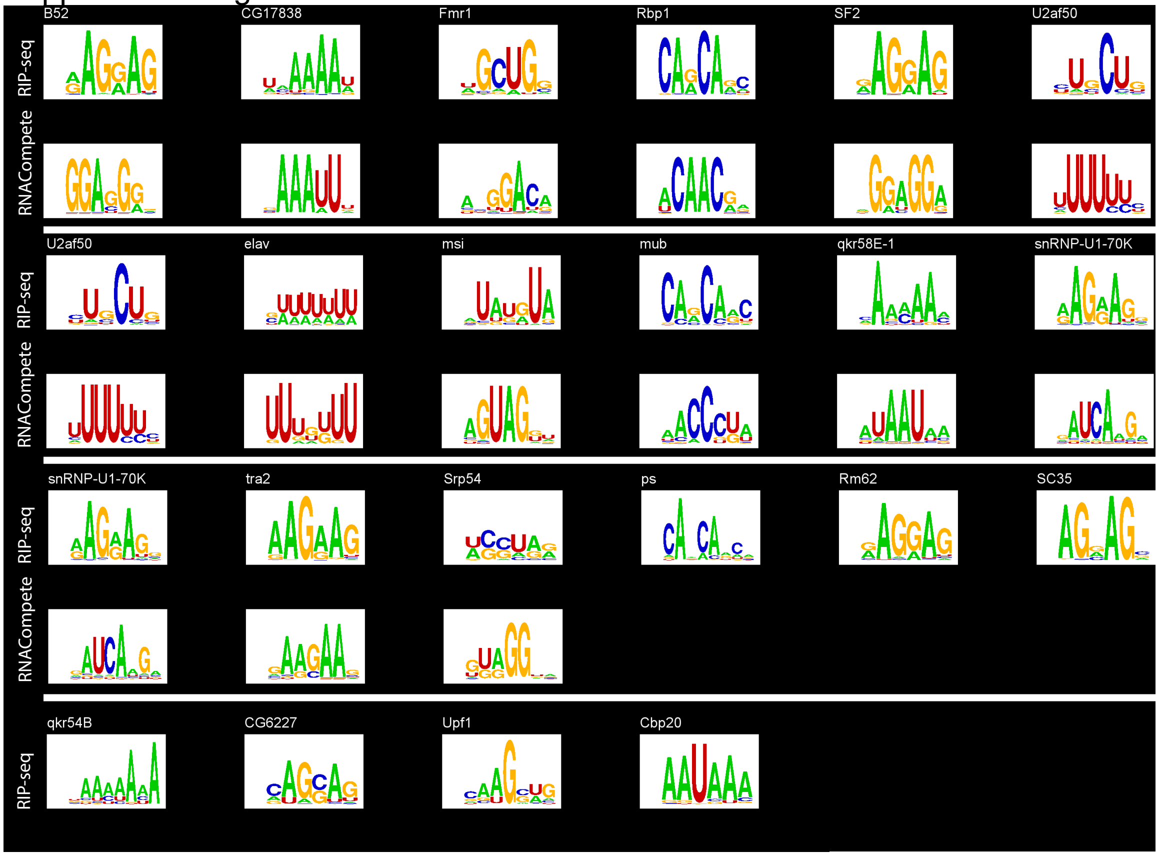

Supplemental Figure 8

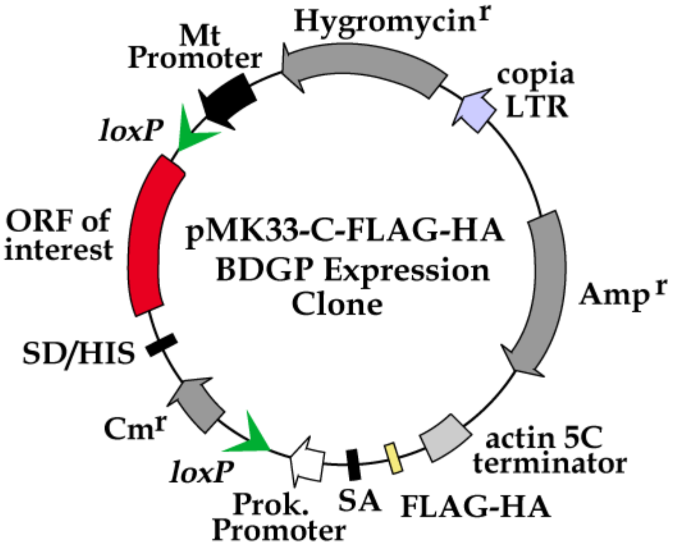

Supplemental Figure 9

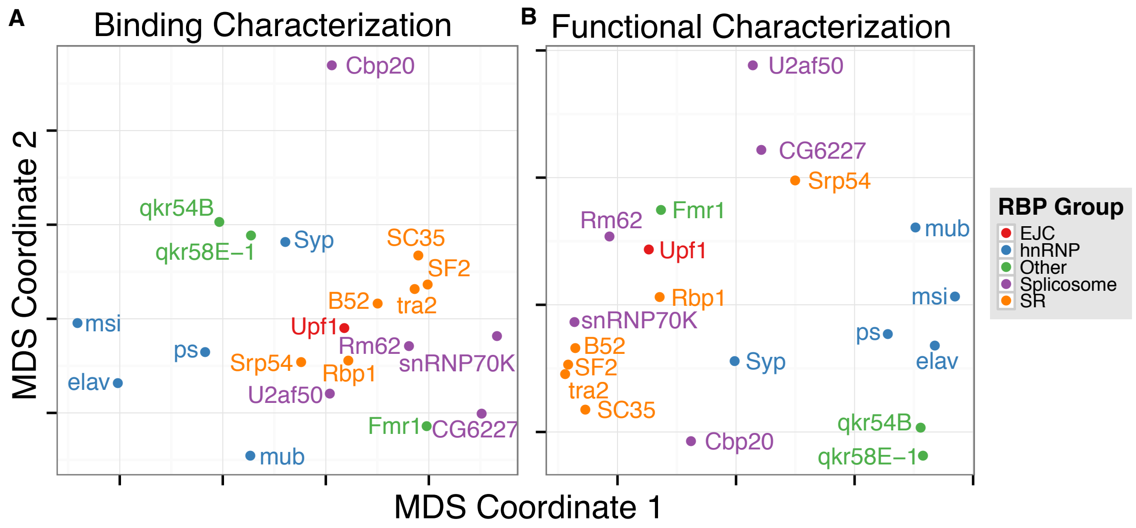

Supplemental Figure 10

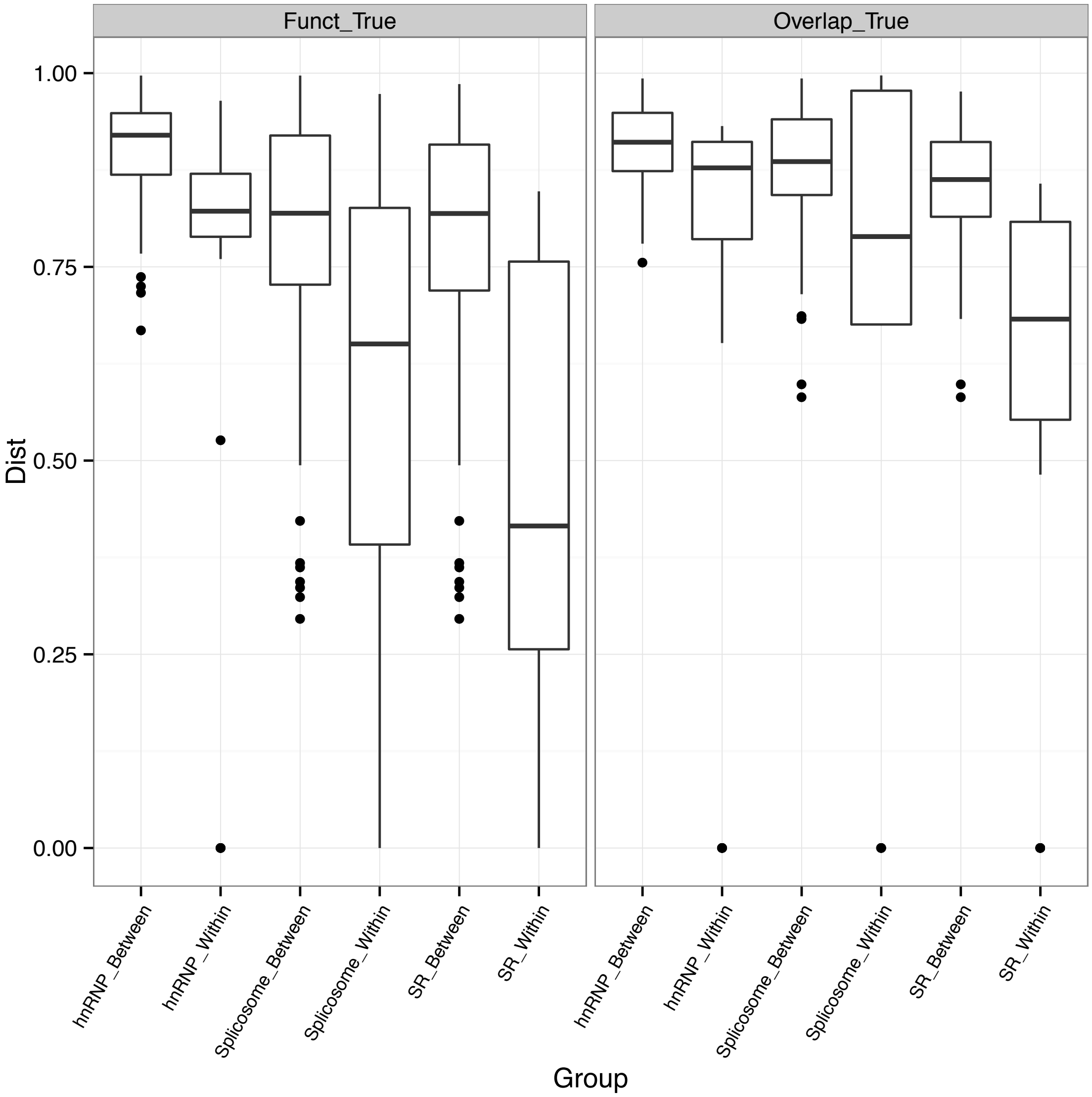

Supplemental Figure 11

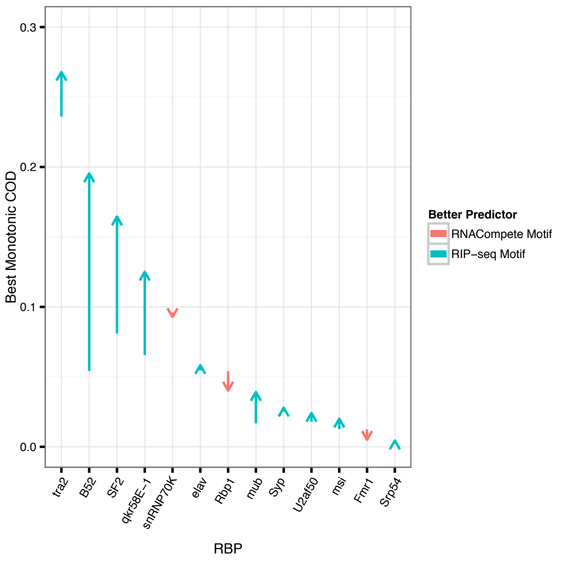

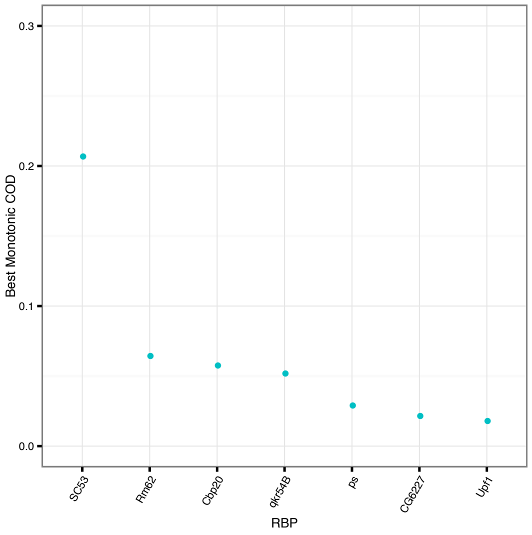

Supplemental Figure 12

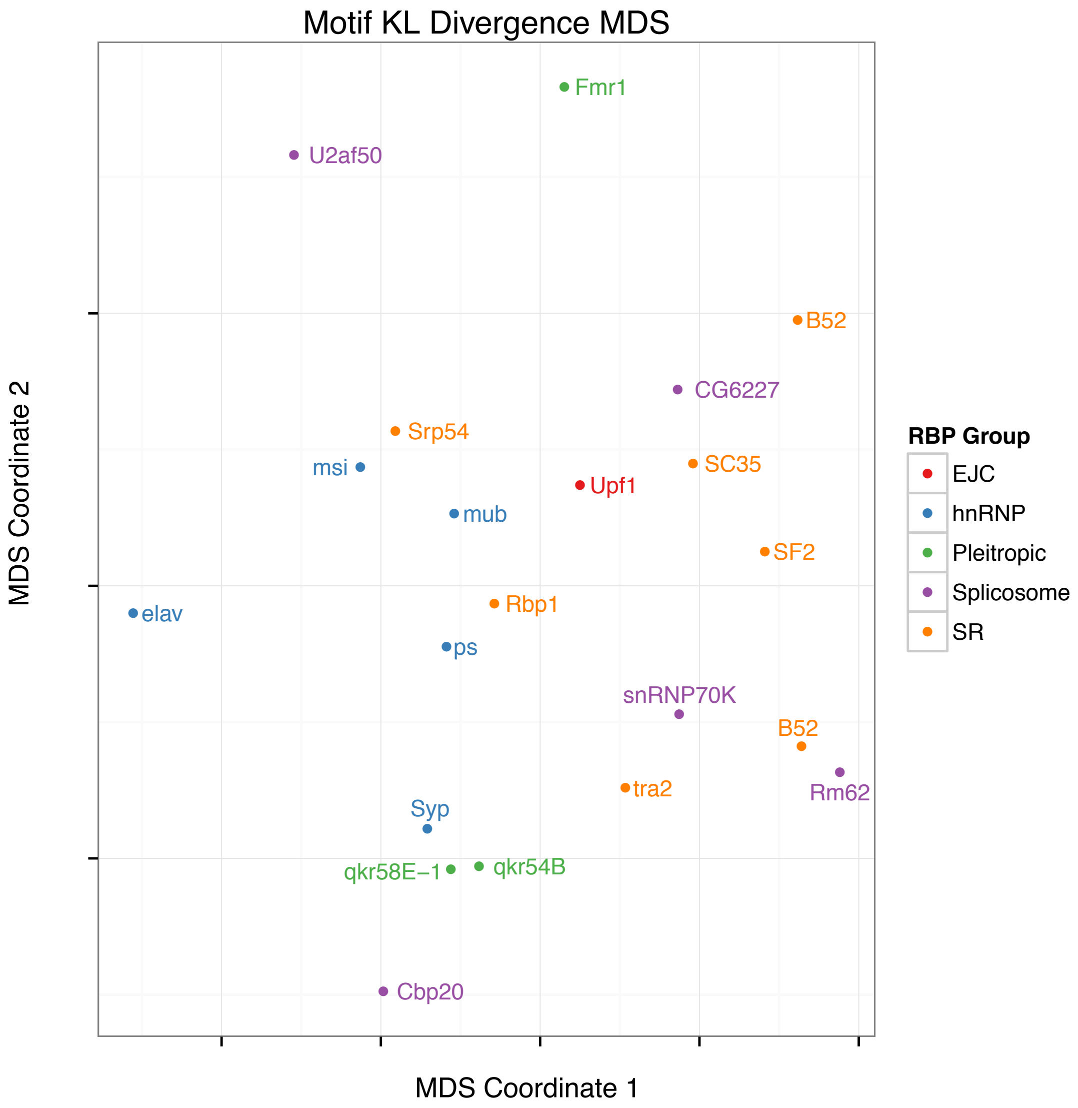

Supplemental Figure 13

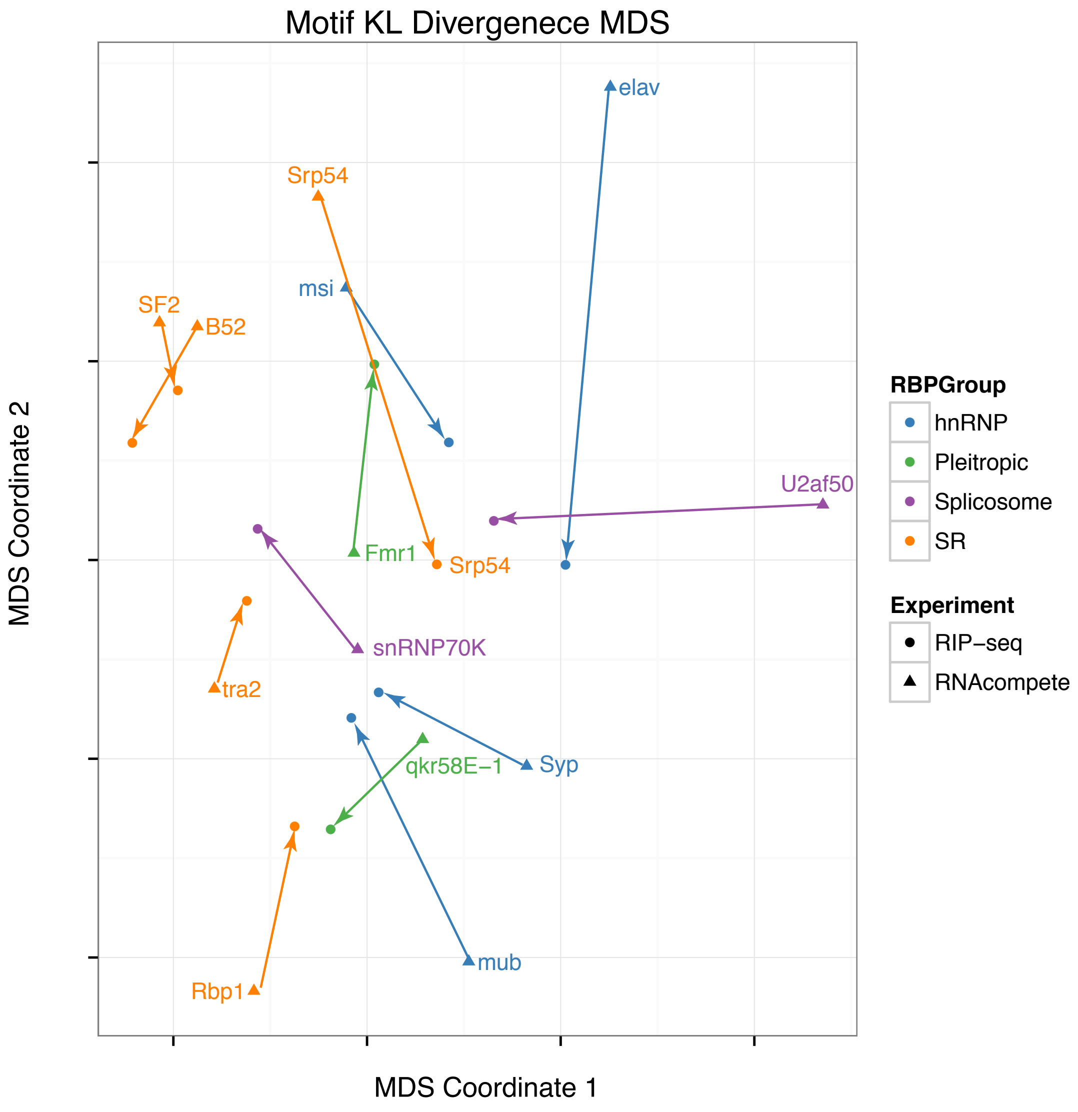

Supplemental Figure 14

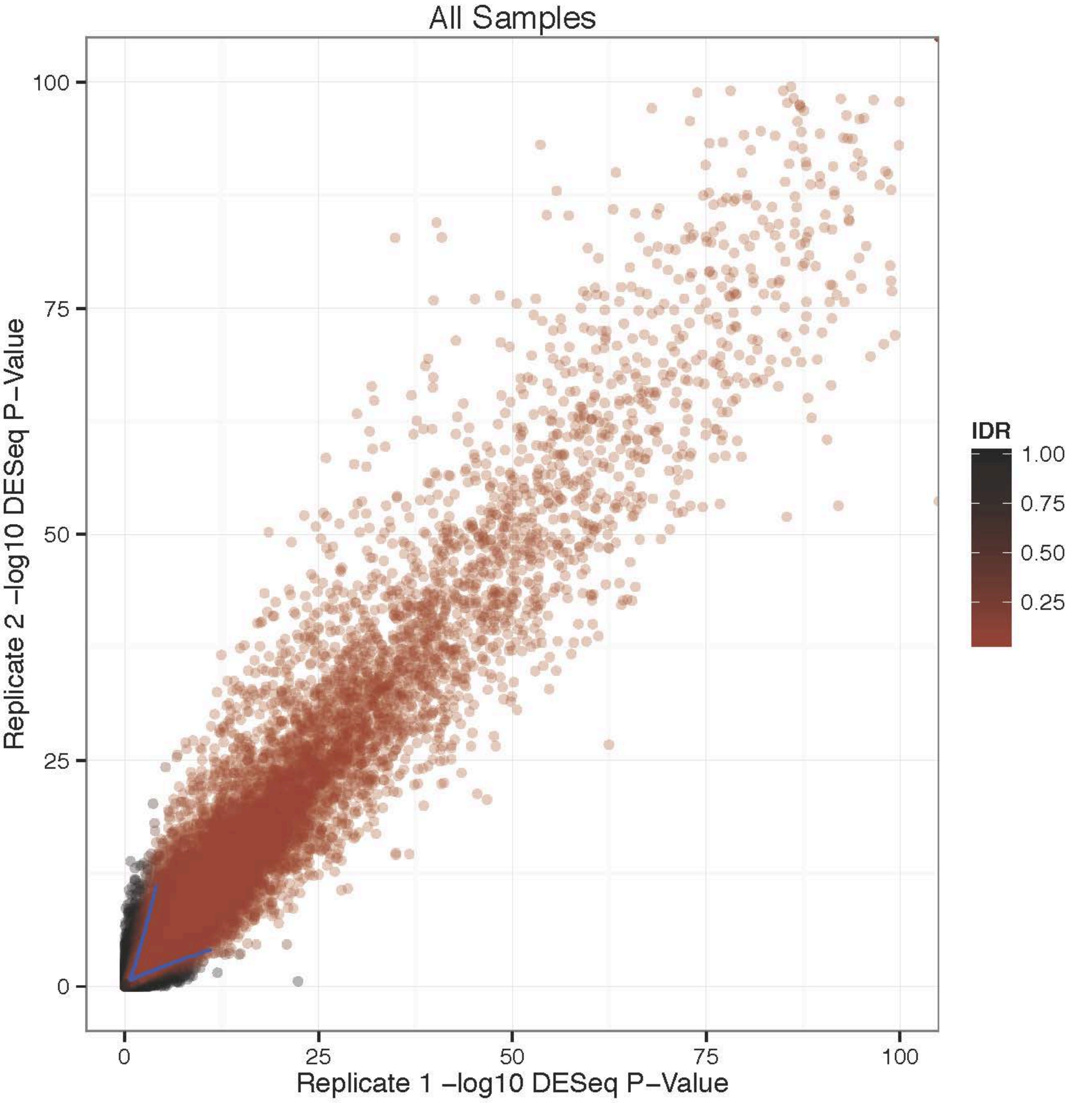

Supplemental Figure 15

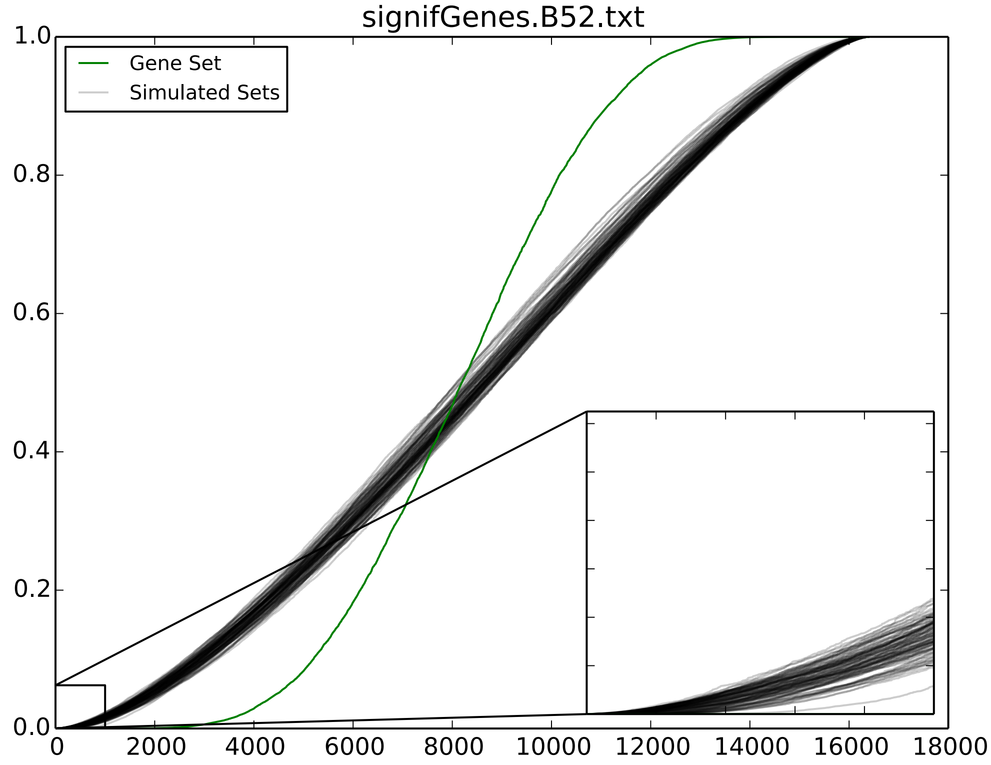

Supplement: Supplemental Material [file supp_gr.182675.114_SuppFigures.pdf]
